# Alpha-(1,6)-fucosyltransferase (FUT8) affects the survival strategy of osteosarcoma by remodeling TNF/NF-κB2 signaling

**DOI:** 10.1038/s41419-021-04416-x

**Published:** 2021-12-02

**Authors:** Shanyi Lin, Lenian Zhou, Yang Dong, Qingcheng Yang, Quanjun Yang, Hanqiang Jin, Ting Yuan, Shumin Zhou

**Affiliations:** 1 Department of Orthopaedic Surgery, Shanghai Jiao Tong University Affiliated Sixth People’s Hospital, Shanghai, China; 2 Department of Pharmacy, Shanghai Jiao Tong University Affiliated Sixth People’s Hospital, Shanghai, China; 3 Institute of Microsurgery on Extremities, Shanghai Jiao Tong University Affiliated Sixth People’s Hospital, Shanghai, China

**Keywords:** Glycobiology, Bone cancer, Apoptosis

## Abstract

Glycosylation is an important modification of membrane proteins that results in functional changes in many cellular activities, from cell-cell recognition to regulatory signaling. Fucosyltransferase 8 (FUT8) is the sole enzyme responsible for core fucosylation, and aberrant fucosylation by dysregulated expression of fucosyltransferases is responsible for the growth of various types of carcinomas. However, the function of FUT8 in the progress of osteosarcoma (OS) has not been reported. In this study, we found that FUT8 is expressed at lower levels in patients with OS and in human OS cell lines such as MNNG/HOS, U2OS, and 143B, suggesting that attenuated expression of FUT8 is involved in the growth and progression of OS. Mechanistically, FUT8 affects the survival strategy of OS by modifying core-fucosylation levels of TNF receptors (TNFRs). Lower fucosylation of TNFRs activates the non-canonical NF-κB signaling pathway, and in turn, decreases mitochondria-dependent apoptosis in OS cells. Together, our results point to FUT8 being a negative regulator of OS that enhances OS-cell apoptosis and suggests a novel therapeutic strategy for treating OS.

## Introduction

Osteosarcoma (OS) is the most frequent and aggressive primary malignant neoplasm diagnosed in the skeletal system [1]. Many patients have to face death threat after diagnosis due to its rapid progression, tendency to metastasize to the lungs, and high recurrence rate [2]. Since the advent of a comprehensive therapeutic strategy in the 1970s, which includes neoadjuvant chemotherapy, surgery, and radiotherapy, the 5-year overall survival rate increased for localized OS [1]. However, the survival rate has remained unchanged for decades, leaving 30–40% of patients who still cannot benefit from comprehensive therapy [3]. Therefore, it is important to identify all the factors that contribute to OS progression. Identification of such factors could pave the way for the development of novel clinical treatments for OS.

Glycosylation is one of the most important post-translational modifications of proteins in all cells [4], as it plays an indispensable role in maintaining many biological functions of proteins. Aberrant glycosylation is closely linked to a variety of diseases, especially cancers, and is due to alternations in the expression of glycosyltransferase [5]. Aberrant glycosylation affects well-known intrinsic traits of tumors, such as proliferation, metastasis, angiogenesis, and drug resistance, among others [6–9]. Fucosylation is a significant kind of glycosylation that is catalyzed by fucosyltransferases (FUTs) [10]. FUTs can be divided into four types according to their catalytic products: FUT1-2, FUT3-7, FUT9-11, and FUT8 [11, 12]. FUT8 exclusively catalyzes the formation of α-1,6 fucosylation—also known as core fucosylation—by connecting fucose to the inner GlcNAc residue of *N*-glycans [11, 13].

FUT8 has been extensively investigated in various cancers. Recent studies indicate that FUT8 expression levels are highly correlated with cancer development, in particular, hepatocellular carcinoma [14], melanoma [15], breast cancer [16], and gastric cancer [17]. Whether FUT8 might also influence the progression of OS is unknown. Furthermore, it is unclear what the underlying mechanism could be.

In the present study, we found significantly lower expression of FUT8 in human OS tissues compared to normal healthy bone tissue. We also found that the expression of FUT8 was negatively correlated with growth of human OS cell lines. In vitro and in vivo assays confirmed that the downregulation of FUT8 in OS cells promoted the progression of OS. Transcriptome sequencing analysis, cell biology experiments and signaling pathway validation provided strong evidence for a possible mechanism. When core fucosylation of TNF receptors was downregulated in OS cell lines due to knockdown of FUT8, the TNF/NF-κB2 signaling pathway was activated and mitochondria-dependent apoptosis was blocked. Together, our findings highlight the important role of FUT8 in the oncogenesis of OS and suggest a new therapeutic strategy for inhibiting OS by changing its glycosylation profile, especially core fucosylation.

## Materials and methods

### Clinical samples

All OS tissues and normal cancellous bone tissues were collected from 2014 to 2015 from the Department of Orthopaedic Oncology, Shanghai Jiao Tong University Affiliated Sixth People’s Hospital. All patients were pathologically diagnosed with osteosarcoma and underwent primary OS resection. Immediately after excision, the samples were frozen in liquid nitrogen to prevent protein and RNA degradation and then stored at −80 °C. Ethics approval was obtained from the Shanghai Sixth People’s Hospital Ethics Committee (YS-2016-064, 24 February 2016).

### Cell lines

The human OS cell lines MNNG/HOS (short for MNNG in the following description), U2OS, and 143B and human osteoblast cell line hFOB1.19 were acquired from American Type Culture Collection (ATCC, USA). OS cell lines MNNG and 143B were cultured in DMEM (Corning, USA) at a 37 °C atmosphere containing 5% CO_2_. OS cell line U2OS was cultured in RPMI 1640 (Corning, USA) at a 37 °C atmosphere containing 5% CO_2_. Osteoblast cell line hFOB1.19 was cultured in D-MEM/F-12 (Gibco, USA) at a 33.5 °C atmosphere containing 5% CO_2_. All culture mediums were supplemented with 10% fetal bovine serum (Gibco, USA).

### Stable cell line construction

The lentivirus shuttle plasmids containing full-length FUT8 (for overexpressing FUT8) and siRNA against FUT8 (for knockdown FUT8) (shFUT8-2 was selected according to efficiency) were co-transfected into HEK293T cells with lentivirus packing vectors, respectively. After 48 h, the supernatant of HEK293T, which containing lentivirus were collected, purified and performed titer determination. Then, MNNG cells were infected by the collected lentivirus at the MOI = 10.0. The positive cells were selected by puromycin at the concentration of 1.0 μg/ml after 72 h after infection. Finally, the stable cells overexpressing (MNNG-F8) and knock down FUT8 (MNNG-siF8) gene were verified by RT-qPCR and WB at both mRNA and protein levels. All the information of the vector and sequences of full-length FUT8 and siRNA against FUT8 has been provided in the Supplementary Table. 1.

### Total RNA extraction and Real-time Quantitative PCR

Trizol reagent (Invitrogen, USA) was used to extract total RNA from clinical samples and cell lines. Reverse transcription was achieved by a RevertAid First Strand cDNA Synthesis Kit (Invitrogen, USA). Revsese transcription quantitative PCR (RT-qPCR) assays were carried out on an ABI Prism 7900HT real-time system (Applied Biosystems, USA) by applicating specifically primers and the SYBR gene PCR master mix (Invitrogen, USA). The 2^−ΔΔCt^ approach was used to calculate the relative mRNA expression of different genes. All primers are shown in Supplementary Table 2.

### Western blot and reagent

RIPA solution (EpiZyme, PRC) containing proteinase inhibitor (Invitrogen, USA) was added to culture cells to gain lysis solution. Then the lysates were centrifuged (13000 g/15 min, 4 °C) and discarded precipitate. Equal amounts of collected total proteins were separated by SDS-PAGE and transferred to a PVDF membrane. Giving the membranes a dip in 5% milk at room temperature (RT) for 1 h. Then, proteins were detected by incubated membranes in primary antibodies solution at 4 °C overnight. After that, HRP-linked anti-IgG antibodies were employed as secondary antibodies. Primary antibodies were shown in Supplementary Table. 3.

### Real Time Cellular Analysis (RTCA) and colony formation assay

The cell proliferation ability was measured by RTCA (ACEA Biosciences, USA) according to the manufacturer’s instructions. Particularly, first, 100 μl of culture medium was added to wells and incubation at 37 °C in a cell incubator for 1 h. Then the baseline value was measured by the incubated medium. Finally, the cells were seeded into wells, and make sure that there were 2.5 × 10^3^ cells per well. Cell attachment and cell proliferation were continuously recorded for 6 h and 168 h, respectively. Cell index was used to representing cell attachment and cell proliferation [18]. For colony formation ability detection, cells were incubated in a 6-wells plate for 2 weeks at a density of 1 × 10^3^ cells per wells. After being fixed with 4% paraformaldehyde (PFA), the cells were stained with crystal violet for 30 min. The colonies which contain over 50 cells were recorded and counted by image J software.

### Subcutaneous tumor model

Four- to five-week-old female nude mice were raised in the Laboratory Animal Research Centre of Shanghai Sixth People’s Hospital. All experiments were approved by the Animal Research Committee of Shanghai Sixth People’s Hospital. For the subcutaneous xenograft tumor model, ten mice were randomly assigned to two groups (*N* = 5) and anesthetized by intraperitoneal injection of 1% pentobarbital sodium at a dose of 0.01 ml/g. Then, the cells were harvested from cell culture flasks and resuspended in PBS to a final concentration of 5 × 10^6^/ml. After that, 200 μl cell suspension containing 1 × 10^6^ cells were injected into the nude mouse flank [19]. Researchers blinded to group allocation measured the tumors with callipers until the longest diameter of the largest tumor reached 200 mm, and all tumors were extracted via surgery. The volume of tumors was calculated as the length (mm) × width (mm)^2^.

### Cell apoptosis analysis

Cells were cultured in a 6-wells plate. After being treated with 0.05 μM staurosporine (STS) (Sigma, USA) for 24 h [20], cells were washed with a cold Phosphate Buffered Saline (PBS) solution followed by digested with trypsin. Digested cells were processed by the Annexin V-PI kit (Beyotime, PRC) according to the manufacturer’s instructions. The result was measured by flow cytometry.

### Mitochondrial membrane potential assay

Cells were cultured in a 6-wells plate. After being treated with 0.05 μM STS for 24 h, cells were washed with a cold PBS solution. Cells were processed by mitochondrial membrane potential assay kit (Beyotime, PRC) according to the manufacturer’s instructions. The images were taken by DM6B fluorescence microscope (Leica, BRD).

### RNA-seq and analysis

Total RNA was extracted from MNNG-siF8, MNNG-F8, and control cell lines by Trizol reagent (Invitrogen, USA). The RNA-Seq libraries were synthesized by the TruSeq™ RNA Sample Preparation Kit (Illumina, USA) following standard Illumina guidelines. After purification, the quantification and validation of the libraries were performed by Qubit® 2.0 Fluorometer (Life Technologies, USA) and Agilent 2100 bioanalyzer (Agilent Technologies, USA), and finally, the library was sequenced by Illumina NovaSeq 6000 (Illumina, USA). The library construction and sequencing were performed by Sinotech Genomics Co., Ltd (Shanghai, PRC). The selection criteria of differential expressed genes (DEG) was a less than five percentage false discovery rate (FDR) and changed expression higher than 1.5 or lower than 0.67 folds. All cell lines were tested three times. The raw RNA-seq data was uploaded to NCBI SRA database. The SRA accession number: PRJNA772908.

### Cell nucleus isolation

For detection of the nuclear translocation of p52, the nucleus was isolated for western blot analysis. Cells were cultured in a 100 mm dish, and nuclei were extracted with a Nuclei EZ Prep kit (Sigma–Aldrich, USA). Briefly, the cells were cultured in a 100 mm diameter cell culture dish. When the cells grew to a density of 80–90% (approximately 1 × 10^7^ cells), they were harvested and lysed by adding 4 ml of ice-cold Nuclei EZ lysis buffer to the cell dish. Then, the lysates were centrifuged at 500 g for 5 min at 4 °C, and the nuclei were resuspended and washed in 4 ml of ice-cold Nuclei EZ lysis buffer. Centrifugation was performed again at 500 g for 5 min at 4 °C, and the nuclei pellet was resuspended in 200 µl of ice-cold Nuclei EZ storage buffer and stored at −70 °C. Then, the expression of p52 was detected in both the nucleus and cytoplasm. PCNA and GAPDH were used as internal references.

### Immunofluorescence (IF) analysis

Cells were cultured in coverslips which were placed in a 24-wells plate. When confluent up to 30%, cells were fixed with 4% PFA for 20 min at RT. After being washed with PBS, fixed cells were blocked by incubating in QuickBlock™ (Beyotime, PRC) for 1 h. Then, cells were washed by PBS three times and incubated with rabbit anti-NF kappaB p100/p52 (1:200, AF6373, Affinity, PRC) overnight at 4 °C. Subsequently, discarded primary antibody and washed by PBS three times, cells were incubated with Alexa Fluor 488-conjugated goat anti-rabbit antibody at RT for 1 h. PI was used to stain nuclei. Images were taken by Confocal Microscope (Leica Microsystems, BRD).

### Immunohistochemical (IHC) analysis

The clinical sample tissues were obtained from Shanghai Jiao Tong University Affiliated Sixth People’s Hospital, and the OS tissues were obtained from subcutaneous tumor model. Both clinical tissues and excised OS tissues were embedded with paraffin. Cutting 4μm sections from the paraffin-embedded tissue sample and deparaffinized. After antigen retrieval, the samples were blocked with 5% bovine serum albumin (BSA) at 37 °C for 30 min. Then, samples were incubated with primary antibodies overnight at 4 °C. After that, samples were washed by PBS and incubated in HRP-linked anti-IgG at 37 °C for 30 min. Three times PBS washing was performed again and 3,3′-diaminobenzidine (DAB) was added to stain for 10 min. Finally, undergo counterstained and dehydrated, samples could be covered by cover glass and photographed by DM6B (Leicag, BRD).

### Immunoprecipitation (IP) and lectin blot

IP was performed in the MNNG-siFUT8, MNNG-FUT8, and control cell lines using a Pierce coimmunoprecipitation kit (26149, Invitrogen, USA) and anti-TNFR antibody (Affinity, AF0282). Briefly, 20 μg of the TNFR antibody (Affinity, PRC) was incubated with the AminoLink Plus coupling resin for 120 min at room temperature and covalently coupled. During incubation, the protein lysates of cells were extracted by adding 1 ml cold IP lysis/wash buffer to the culture dish (100 × 100 mm). Then, the antibody-coupled AminoLink Plus coupling resin was washed and incubated with 200 µl of the whole cell lysates overnight at 4 °C. Subsequently, the resin was washed again, and the proteins bound to the antibody were eluted by elution buffer. The core fucosylation of the TNF receptor was examined by lectin blot using Aleuria aurantia lectin (AAL) (B-1395, Vectorlabs, USA) from the extracted proteins. The extracted proteins were separated by SDS–PAGE and transferred to a PVDF membrane. The membranes were dipped in 5% milk at room temperature (RT) for 1 h. Then, the proteins were detected by incubating membranes in AAL solution at room temperature for 2 h. Subsequently, HRP-linked avidin was employed as a secondary antibody.

### Statistical analyses

All data were analyzed by SPSS 25.0 software and presented as the mean ± SD. The two-tailed Student’s t-test was performed to calculate the differences between the experimental group and the control group. The welch’s t-test was performed to calculate the differences between the tumor tissues group and non-tumor tissues group of clinical samples. ns means *P* > 0.05, * means *P* < 0.05, ** means *P* < 0.01, *** means *P* < 0.001.

## Results

### Reduced expression of core fucosyltransferase FUT8 in human clinical OS samples and OS cell lines

**Fig. 1:**
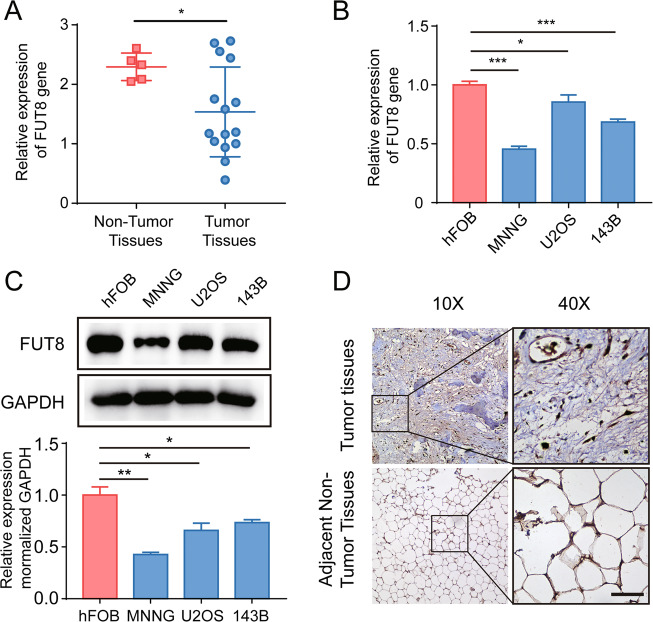
Expression of FUT8 is reduced in osteosarcoma tissue and osteosarcoma cell lines. **A** Relative expression of *FUT8* mRNA in human osteosarcoma (OS) tissue samples (*N* = 15) and adjacent tumor-free cancellous bone tissue samples (*N* = 5). Individual qRT-PCR values of each sample are superimposed on the group mean and SD bars. **B** Mean relative ±SD (*N* = 3) expression of *FUT8* mRNA in OS cell lines (MNNG, U2OS, 143B) and the osteoblast cell line hFOB1.19. **C** Western blot of FUT8 protein (upper panels) and quantitation of protein levels (lower bar graphs) in OS cell lines and hFOB1.19 cell extracts (*N* = 3). **D** Representative images of FUT8 immunohistochemical staining in OS tissue sections and adjacent tumor-free bone marrow tissue sections. Immunostaining was visualized with DAB with peroxidase as the chromogen. Tissue sections are counterstained with hematoxylin (**D** scale bars, 100 μm). *P < 0.05; **P < 0.01; ***P < 0.001.

To examine the role of FUT8 in the progression of OS, we first analyzed the expression of *FUT8* in clinical samples of normal cancellous bone and OS tissue samples by RT-qPCR. *FUT8* mRNA expression was significantly lower in extracts from OS tissue compared to extracts from adjacent normal bone (Fig. 1A). In OS cell lines (MNNG, U2OS, and 143B) and an osteoblast cell line hFOB1.19, we quantified both *FUT8* mRNA and protein levels using qPCR and western blotting. OS cell lines had lower levels of FUT8 expression compared to that in the osteoblast cell line hFOB1.19 (Fig. 1B, C). We also examined the distribution of FUT8 by immunohistochemical staining OS tissue sections and adjacent non-tumor sections. Consistent with the RT–qPCR results, visual observation of FUT8 immunostaining indicated that it was less dense in OS tissue sections than in nearby normal bone marrow tissue (Fig. 1D). Taken together, these results suggest that downregulation of *FUT8* may be related to tumorigenesis in OS.

### FUT8 negatively regulates the progression of OS in vitro and in vivo

Since there was significantly reduced expression of FUT8 in human OS tissue and cell lines, we hypothesized that FUT8 negatively regulates the progression of OS. To test this idea, we constructed two FUT8-regulated stable OS cell lines: MNNG-F8, which overexpresses FUT8, and MNNG-siF8, which knockdown FUT8. Both of these lines, as well as their control cell lines, were constructed using lentivirus.

**Fig. 2:**
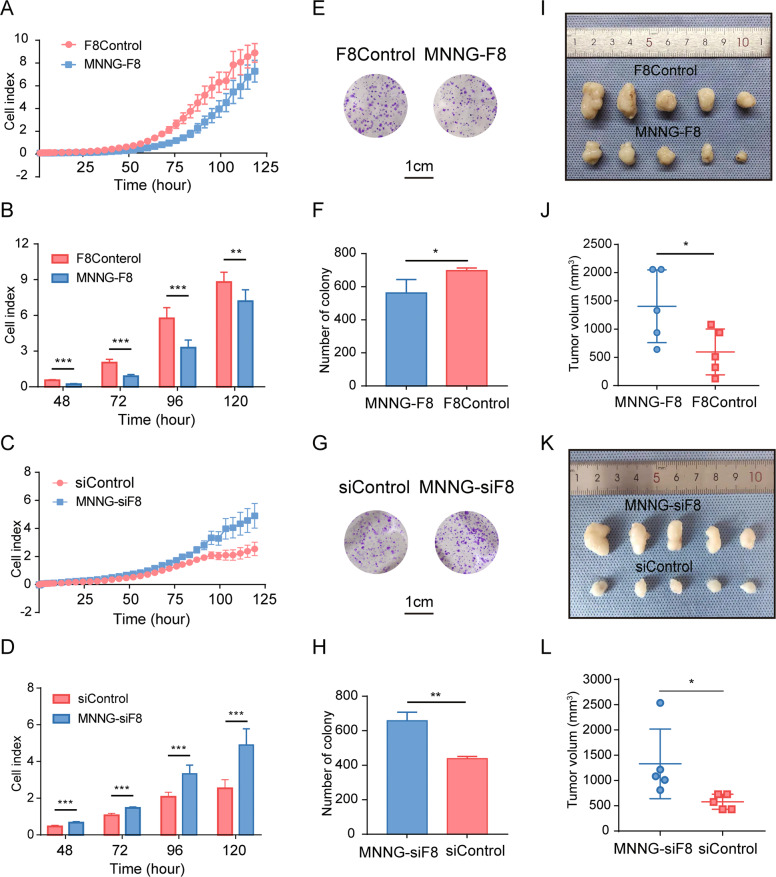
FUT8 expression is negatively correlated with OS oncogenesis in vitro and in vivo. **A**, **C** Mean ± SD (*N* = 4) cell index, a measure of cell attachment and cell proliferation in vitro, for two FUT8-regulated OS cell lines (MNNG-F8, MNNG-siF8) and their control cell lines (F8Control, siControl, respectively) over time. RTCA assay confirmed that cell proliferation is enhanced in vitro in MNNG-siF8 cells, while it is reduced in MNNG-F8 cells compared to control cells. **B**, **D** Mean ± SD (*N* = 4) cell index at 48 h, 72 h, 96 h, 120 h. **E**, **G** Representative light microscopic images of PFA-fixed MNNG-F8, MNNG-siF8, and control cells stained with crystal violet, and (**F, H**) quantitation of colony counts after 2 weeks of in vitro maintenance, data were shown as mean ± SD (*N* = 3). **I**, **K** Images of excised flank tumors after injecting 200 μl of cell suspension from MNNG-F8 (*N* = 5), MNNG-siF8 (*N* = 5), or control cells (*N* = 5) in the nude mouse subcutaneous tumor model, and (**J**, **L**) quantitation of tumor volume (mean ± SD). *P < 0.05; **P < 0.01; ***P < 0.001.

Successful overexpression and knockdown of FUT8 was verified when we measured its levels of protein (Fig. S1A, 1B) and mRNA (Fig. S1C, 1D). To examine whether altered expression of FUT8 affects cell function in OS cells, we assessed OS cell growth by Real Time Cellular Analysis (RTCA) [18]. Proliferation was measured in MNNG-F8, MNNG-siF8, and control cell lines. As expected, cell proliferation was significantly reduced in cell lines that overexpressed FUT8. By contrast, cell proliferation was enhanced in cell lines that knockdown FUT8 (Fig. 2A–D). The results from colony formation assays followed the same pattern as the RTCA results. Compared with controls, colony-forming assays showed that colony formation was reduced or increased, respectively, depending on whether FUT8 was over- or under-expressed (MNNG-F8 vs. MNNG-siF8, respectively) (Fig. 2E–H).

The role of FUT8 in OS cell proliferation in vivo was also examined in the nude mice subcutaneous implantation tumor model. Tumors were measured every 3 days until the longest diameter of the largest tumor reached 200 mm. FUT8 overexpression inhibited tumor growth, while FUT8 knockdown enhanced tumor growth (Fig. 2I–L).

We were also interested in the possible role of FUT8 in metastasis in OS. Thus, we measured the migration and invasion characteristics of MNNG-F8 and MNNG-siF8 cells using the RTCA assay. A similar pattern of results was obtained for migration and invasion as for the tumor morphological experiment above. Migration and invasion were enhanced in FUT8-knockdown MNNG-siF8 cells. However, migration and invasion tended to be blunted in FUT8-overexpressing MNNG-F8 cells, but the reduction was not significant (Fig. S2). In summary, these results show that the expression of FUT8 is negatively correlated with cell proliferation and colony formation in OS cells.

### Downregulation of FUT8 inhibits apoptosis in OS cells

**Fig. 3:**
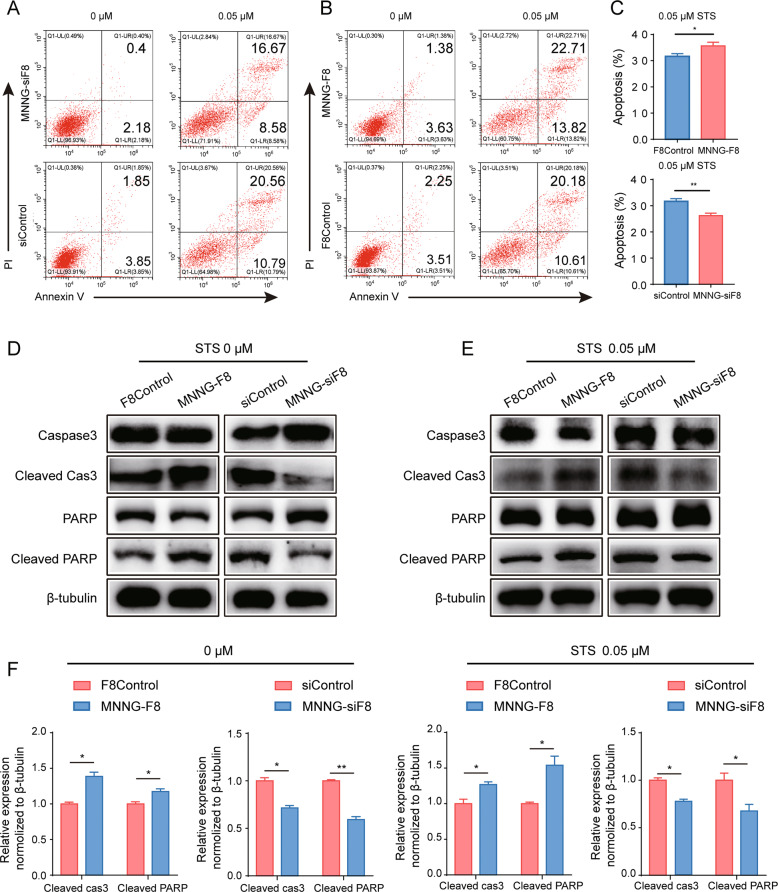
Expression of FUT8 is positively correlated with the apoptotic rate of OS cells. **A**, **B** Two-label dot plots of flow cytometry analysis of two FUT8-regulated OS cell lines (MNNG-F8, MNNG-siF8) and their control cell lines (F8Control, siControl, respectively) treated with either 0 μM or 0.05 μM STS for 24 h. Cells were stained with Annexin V/PI. Compared to control cells, in MNNG-F8 and MNNG-siF8, apoptosis was enhanced and reduced, respectively. **C** Quantitation of flow cytometry analysis, data were shown as mean ± SD (*N* = 3). **D**, **E** Western blot probed with antibodies against apoptosis-related proteins in the four cell lines with or without 0.05 μM STS treatment confirmed that, compared to control cells, cleaved-caspase 3 and cleaved-PARP were more or less abundantly expressed, respectively, in MNNG-F8 and MNNG-siF8 cells. **F** Quantitative expression values of the WB results in D and E were normalized to β-tubulin expression (*N* = 3). *P < 0.05; **P < 0.01.

We next examined potential mechanism(s) for FUT8 in OS cell growth. During in vitro maintenance of our cell lines, it became apparent that apoptotic rates in MNNG-siF8 were reduced (data not shown). Thus, we measured cell apoptosis with Annexin V/PI staining in MNNG-siF8, MNNG-F8 and their control cell lines treated with or without 0.05 μM STS, and then we identified cell types with flow cytometry. Flow cytometry revealed a significantly enhanced apoptosis phenotype in the MNNG-F8 group (36.53% VS 30.79%) and a significantly depressed apoptosis phenotype in the MNNG-siF8 group (25.25% VS 31.15%), as compared to control cells (Fig. 3A–C). Western blot analysis of apoptosis-related proteins in MNNG-siF8, MNNG-F8 and their control cell lines treated with or without 0.05 μM STS confirmed that, the level of FUT8 expression is related to apoptosis. Regardless of the treatment with or without 0.05 μM STS, the levels of cleaved-caspase 3 and cleaved-PARP in MNNG-siF8 cells were significantly reduced while the levels of these two proteins in MNNG-F8 cells were increased (Fig. 3D–F). Together, these results support the hypothesis that FUT8 regulates OS tumor growth through cell apoptosis.

### FUT8-related apoptosis regulation in OS cells occurs through a mitochondria-dependent apoptotic pathway

**Fig. 4:**
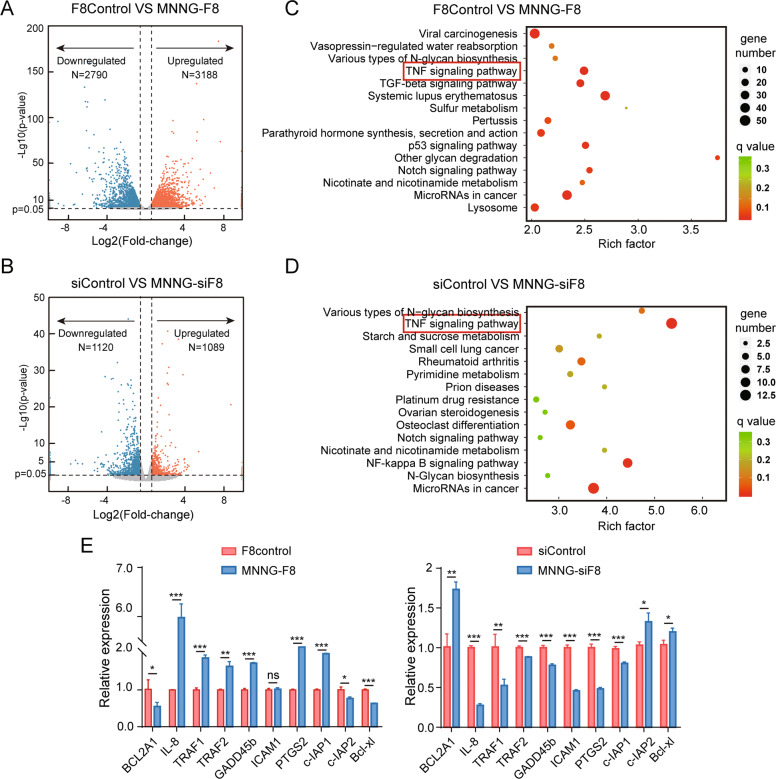
Transcriptome analysis of FUT8-overexpressing MNNG-F8 and FUT8-under-expressing MNNG-siF8 cells and their control cells. **A**, **B** Volcano plots show the differentially expressed genes (DEGs) between the two FUT8-regulated cells and their controls. Fold-change values on the abscissa were log2-transformed, and *P* values on the ordinate were -log10 transformed. The selection criteria of differential expressed genes (DEG) was *P*-value < 0.05 and changed expression higher than 1.5 or lower than 0.67 fold. **C**, **D** KEGG pathway enrichment analysis for DEGs between the two FUT8-regulated cells and their controls. **E** Mean ± SD (*N* = 3) relative expression of DEGs. Among the DEGs, genes that are highly related to the TNF/NF-κB2 pathway were verified by RT-qPCR to be expressed in the FUT8-altered cells and their controls. ns På 0.05; *P < 0.05; **P < 0.01; ***P < 0.001.

In order to examine a possible mechanism of how FUT8 is involved in cell apoptosis, we identified differentially expressed genes (DEGs) via transcriptome sequencing (RNA-seq). This information can help uncover how FUT8 might regulate oncogenesis in OS. RNA-seq analysis revealed a different gene expression profile between control and engineered FUT8-regulated OS cells (Fig. S3A, 3B). These DEGs are shown in Fig. 4A, B. There were 5,978 DEGs between the control and the FUT8-overexpressing cell line, whereas there were 2,209 DEGs between the control and the FUT8-knockdown cell line.

Kyoto Encyclopedia of Genes and Genomes (KEGG) analysis was carried out among the DEGs. Besides the various types of genes associated with the *N*-glycan biosynthesis pathway, genes associated with the highly related pathways of TNF signaling pathway were identified in both MNNG-F8 and MNNG-siF8 cells (Fig. 4C, D). We examined the expression of primary genes related to TNF/NF-κB2 signaling pathways via RT-qPCR in the cell lines. These results were consistent with the RNA-seq results (Fig. 4A, B). As Figs. 4E, S3C and S3D show, *TRAF1/2*, *Gadd45b*, *c-IAP1*, *IL-8*, *ICAM-1*, *PTGS2*, *MAP3K5*, *NFKBIA*, *RIPK2*, and *NGFR* were upregulated while *BCL2A1*, *c-IAP2*, *Bcl-xl*, *TNFSF12*, and *MAP3K14* were downregulated in FUT8-overexpressing cells (MNNG-F8). We observed the opposite expression pattern for these genes in the FUT8-knockdown cells (MNNG-siF8).

**Fig. 5:**
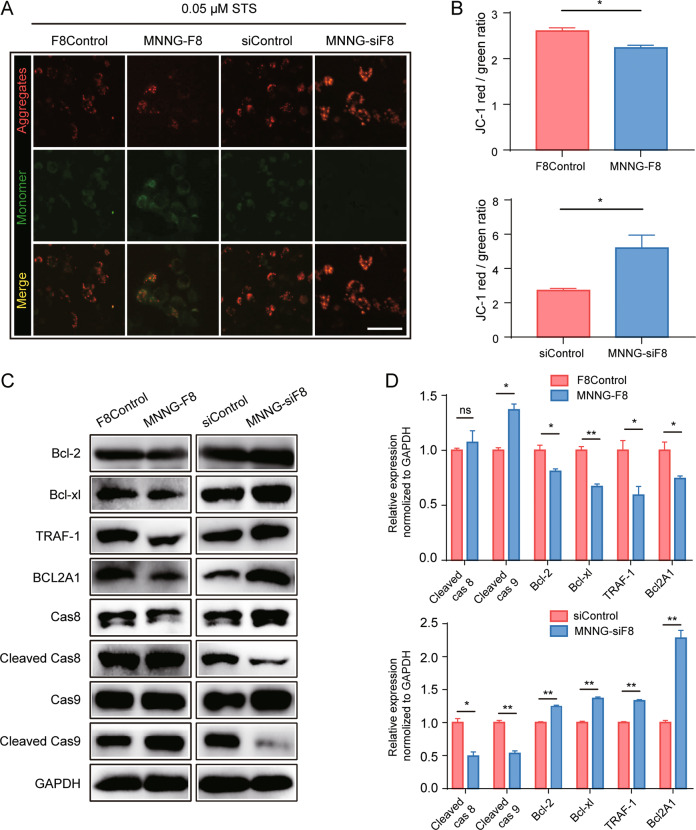
Apoptosis in FUT8-regulated OS cells occurs through a mitochondria-dependent pathway. **A** Representative immunofluorescent images of JC-1 staining in the four OS cell lines after treatment with 0.05 μM STS for 24 h. High ratios of red/green fluorescence represented a high mitochondrial transmembrane potentials (ΔΨM) while low ratios of red/green fluorescence represented a low ΔΨM (Scale bar: 25 μm). **B** Mean ± SD (*N* = 3) ratios of JC-1 red/green fluorescence. **C** Western blot of mitochondria-dependent apoptosis-related proteins (Bcl-2, Bcl-xl, Bfl-1, cleaved-caspase 8, and cleaved-caspase 9) confirmed that, compared to control cells, anti-apoptosis proteins were less abundant in MNNG-F8 cells but were more abundant in MNNG-siF8 cells. Relative expression was normalized to GAPDH. **D** Quantitation of relative expression (mean ± SD, *N* = 3) of mitochondria-dependent apoptosis-related proteins. ns, På 0.05; *P < 0.05; **P < 0.01.

The DEGs we identified were highly related to mitochondria-dependent apoptosis. To test whether apoptosis induced by FUT8 overexpression is indeed mitochondria-dependent, we treated the four cell lines with 0.05 μM STS and then stained them for JC-1 in order to detect mitochondrial membrane potential changes (Fig. 5A). JC-1 is cationic dye that is a proxy for mitochondrial transmembrane potentials (ΔΨM) in healthy and apoptotic cells across multiple cell types [21]. The intensity of green fluorescence (JC-1 monomer) was visibly stronger in MNNG-F8 cells, while red fluorescence (JC-1 aggregates) was stronger in MNNG-siF8 cells compared to controls. The lower ratio of red/green fluorescence (Fig. 5B) found in MNNG-F8 cells indicate a lower mitochondria membrane potential (i.e., impaired mitochondrial function). By contrast, mitochondrial function appeared to be intact in MNNG-siF8 cells (Fig. 5A, B).

Western blot analysis showed that the expression levels of proteins that protected cells from mitochondria-dependent apoptosis (Bcl-2, Bcl-xl, and BCL2A1) were more highly expressed while pro-apoptosis proteins (cleaved-caspase 8 and cleaved-caspase 9) were lower expressed in the MNNG-siF8 cells compared to control cells (Fig. 5C, D). Expression of these proteins in MNNG-F8 cells showed the opposite pattern to that seen in MNNG-F8 cells (Fig. 5C, D). Together, these results support the hypothesis that OS regulated by FUT8 through the mitochondria-dependent apoptotic pathway and can either increase or decrease the cell apoptosis fate of OS cells depending on the expression level of FUT8.

### Downregulation of FUT8 activates the non-canonical TNF/NF-κB signaling pathway

FUT8 regulates OS cells’ apoptosis via a mitochondria-dependent pathway. KEGG analysis revealed that the non-canonical TNF/NF-κB signaling pathway i.e., TNF/NF-κB2 signaling pathways were altered. We examined in more detail whether this signaling pathways are activated and control mitochondria-dependent apoptosis after FUT8 was remodeled in OS cells.

**Fig. 6:**
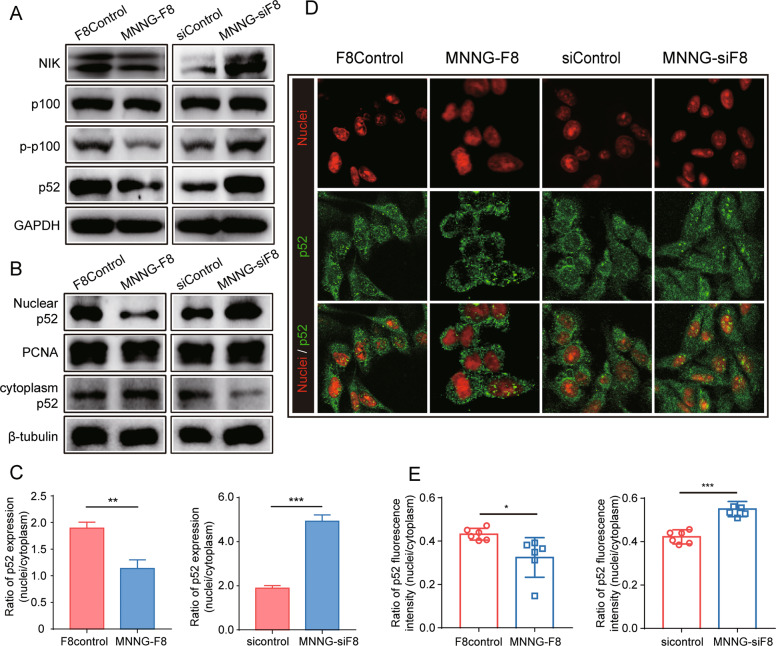
Knockdown of FUT8 in OS cells in vitro activates TNF/NF-κB2 signaling pathway. **A** Western blot of proteins in a NF-κB2 signaling pathway show that this pathway was activated in MNNG-siF8 cells but inhibited in MNNG-F8 cells. **B** Analysis of p52 translocation in OS cell lines. Western blot probed for both in nuclear p52 and cytoplasmic p52. **C** Quantitation of ratio of nuclear p52 to cytoplasmic p52 expression. Mean ± SD (*N* = 3) ratio expression in MNNG-siF8 cells was higher, compared to controls, and lower in MNNG-F8 cells. Relative expression of nuclear p52 was normalized to PCNA while relative expression of cytoplasmic p52 was normalized to β-tubulin. **D** Representative p52 immunofluorescent images of cultured OS cell lines showed translocation of p52 into the nucleus. Red fluorescence represented nuclei, green fluorescence represented p52 (Scale bar: 25 μm). The translocation of p52 into the nuclei appeared visually to be enhanced in MNNG-siF8 cells but reduced in MNNG-F8 cells compared to control cells. **E** Quantitation of ratio of nuclear p52 to cytoplasmic p52 in cultured OS cell lines. Mean ± SD (*N* = 3) ratio of nuclear p52 fluorescence to cytoplasmic p52 fluorescence. *P < 0.05; **P < 0.01; ***P < 0.001.

Previous studies showed that members of the TNFR superfamily could activate the NF-κB2 signaling pathway in immune cells [22, 23]. To examine whether the NF-κB2 signaling pathway might also be activated in OS cells, we measured the expression of key proteins involved in NF-κB2 signaling. As shown in Fig. 6A, the knockdown of FUT8 in MNNG-siF8 cells results in the activation of NF-κB2 signaling pathways. Upregulation of NF-κB-inducing kinase (NIK), led to the phosphorylation of p100 (p-p100) and then degraded into p52. By contrast, the overexpression of FUT8 in MNNG-F8 cells inhibited the activation of NF-κB2 signaling pathways. Since p52 could only play its role as a transcriptional regulator when in nucleus, we next examined the translocation of p52.

Western blot analysis detected the expression of p52 both in the cytoplasm and in isolated nuclei. Compared to control cells, there was more p52 detected in the nucleus of FUT8-knockdown cells and less p52 in the nucleus of FUT8-overexpressing cells (Fig. 6B, C). Translocation of p52 into nucleus was also examined by p52 immunofluorescence staining of cultured OS cell lines. The knockdown of FUT8 significantly enhanced the translocation of p52 into the nucleus. However, the aggregation of p52 in the nucleus was inhibited by FUT8 overexpression (Fig. 6D, E).

### Lack of core fucosylation of TNFR shifts TNF signaling

**Fig. 7:**
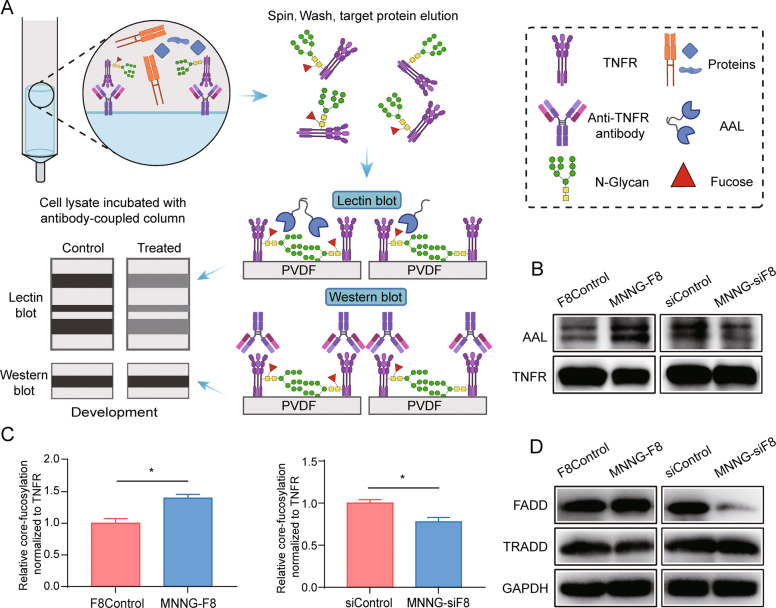
Lack of core fucosylation of TNFR shifts TNF signaling. **A** Diagram illustrating the method for detecting core-fucosylation level of TNFR in FUT8 regulated cells and their according control cells. TNFR was first enriched from the four cell strains by immunoprecipitation using an anti-TNFR antibody. Then, Western blot and lectin blot, respectively, were used to detect expression of TNFR and core-fucosylation level of TNFR. The lectin blot was done using the AAL, which specifically identifies a combination of core-fucosylated structures. Relative expression of core fucosylation was normalized to TNFR expression. **B** Western blot of TNFR and lectin blot of core fucosylation of TNFR. Core fucosylation per unit TNFR was increased in MNNG-F8 but reduced in MNNG-siF8 cells compared to control cells. **C** Quantitation of core-fucosylation levels (*N* = 3). **D** Western blot analysis of the shifts TNF signaling. The TNFR down-stream adaptor molecules that mediates cell apoptotic FADD and TRADD were detected by Western blot. The expression of FADD was obvious changed after altering FUT8 levels, while that of TRADD did not change. Relative expression of proteins was normalized to GAPDH. *P < 0.05.

We were also interested in examining possible changes in signaling upstream of NF-κB2. Figure 7A shows a diagram of the method we used for detecting the relative core-fucosylation level of TNFR in F8Control cells, MNNG-F8 cells, siControl cells, and MNNG-siF8 cells. In particular, we enriched TNFR from the four OS cell lines by immunoprecipitation and detected expression of TNFR. The core-fucosylation level of TNFR was detected by lectin blot. The level of TNFR core fucosylation was reduced in FUT8-knockdown cells but was elevated in FUT8-overexpressing cells (Fig. 7B, C). A question is would the core-fucosylation level influence the function of TNFR?

Western blotting was used to examine the expression of Fas-associated death domain (FADD) and TNF receptor-associated death domain (TRADD), which serve as major downstream factors of TNFR to transduce survival and apoptosis signals [24, 25]. A slightly increased and an obviously decreased expression of FADD was detected in the FUT8-overexpressing and FUT8-knockdown cell lines, respectively. However, we found no apparent differences in the expression of TRADD after the fucosylation level of TNFR was changed (Fig. 7D). In summary, these data, along with the RNA-seq results, support our hypothesis that in OS cells, knockdown of FUT8 reduces core fucosylation of TNFR, causing TNFR to transduce signals via FADD to activate TNF/NF-κB2 signaling pathways.

### FUT8 negatively regulates TNF/NF-κB2 signaling pathway and enhances anti-apoptosis processes in OS

**Fig. 8:**
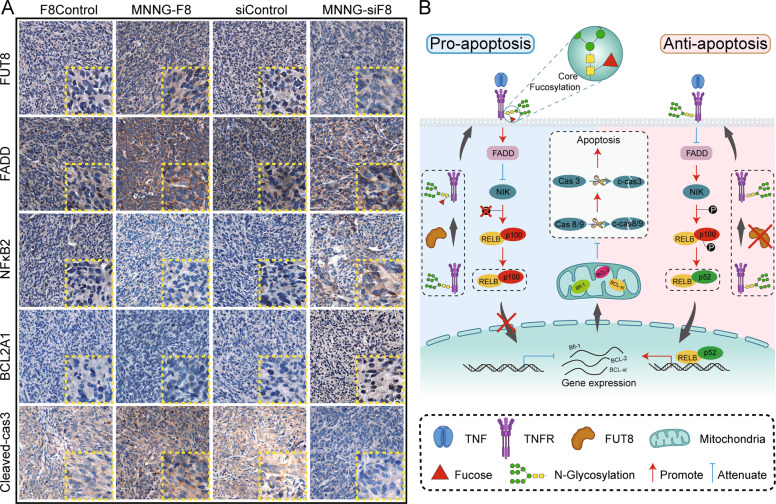
FUT8 negatively regulates NF-κB2 signaling by blocking the activation of TNFR. **A** Immunohistochemical staining of FUT8, FADD, NFκB2, BCL2A1, and cleaved-caspase 3 in xenografts of OS tissue from the subcutaneous tumor nude mouse model visualized by DAB. **B** Diagram illustrating one mechanism that illustrates how FUT8 influences OS cell apoptosis through the TNF/NFκB signaling pathway.

We demonstrated that FUT8 can alter the core-fucosylation level of TNFR, leading to the activation of the TNF/NF-κB2 signaling pathway. These changes finally affect the phenotype of apoptosis. We further examined the regulation of apoptosis in xenografts of OS tissue from the subcutaneous tumor nude mouse model. IHC results confirmed that, compared to control tissue, MNNG-siF8 tissue showed reduced expression of FUT8, FADD, leading to enhanced expression of p52 and Bfl-1 and reduced expression of cleaved-caspase3. There was an opposite expression trend of these key proteins in MNNG-F8 tissue compared to control tissue (i.e., the enhanced expression of FUT8, FADD, and cleaved-caspase3, and the reduced expression of p52 and Bfl-1) (Fig. 8A).

## Discussion

Previous studies demonstrated that in OS, a specific glycosylation profile strongly predicts OS progression. For example, Zhang et al., reported that a glycosyltransferase called *N*-GalNAc transferase is highly expressed in OS [26]. *N*-GalNAc transferase catalyzes mucin-type *O*-glycosylation, a common form of glycosylation in mammals [27]. When the *N*-GalNAc transferase gene is knocked down in OS cells, the invasiveness of OS is significantly reduced [26]. Deng et al. also showed that loss of *O*-GlcNAcylation via degradation *O*-GlcNAc transferase results in the reduction of OS growth [28]. Also, the reduction of galactosyltransferase causes the accumulation of collagen type I in OS, which impared the OS [29]. However, as far as we know, even though the role of FUT8 has been investigated in many cancers such as lung cancer, prostate cancer, and breast cancer [16, 27, 30–32], the relationship between FUT8 and the progression of OS has received no investigative attention before our study.

In our present study, we demonstrated that the expression of FUT8 was low in OS tissues and OS cell lines compared to that in normal controls. We discovered a negative correlation between FUT8 expression and OS proliferation rate. The knockdown of FUT8 in human OS cell lines resulted in reduced core fucosylation of TNFR, which in turn led to the activation of the NF-κB2 signaling pathway. After being translocated into the nucleus, p52, a NF-κB2 family member, remodeled the expression of mitochondria-dependent apoptosis-related proteins, resulting in inhibition of cell apoptosis (Fig. 8B). The TNF/NF-κB2 signaling pathway is a well conserved, and relatively thoroughly investigated pathway [33]. In addition, the idea that glycosylation plays an important role in protein function has been around for decades [34]. However, most studies on the TNF/NF-κB signaling pathway have focused on the binding between ligands and TNFR and on the phenotypic changes of cells caused by the binding [35–38]. To the best of our knowledge, the present study is the first to link the core fucosylation and the function of TNFR that reducing core fucosylation of TNFR via knockdown of FUT8 leads to activation of the TNF/NF-κB2 signaling pathway.

In line with our main finding, Geert et al. recently presented a novel theory that glycosylation serves as a key regulator of TNFR signaling [39]. Their study showed that *N*-glycosylation of TNFR was closely related to TNFR’s ligand-receptor binding ability, and that cells could change their TNFR’s glycosylation profile to “adapt” to different microenvironments [39]. This notion of adjusting glycosylation profile according to the microenvironment is consistent with the findings of Holdbrooks et al., who observed that ST6Gal-I sialyltransferase protects tumor cells from TNF-induced apoptosis [40]. They demonstrated that ectopically overexpressed ST6Gal-I sialylated TNFR, which in turn caused the signal induced by TNF to change from an apoptotic one to a survival one [40]. Our results provide further evidence to support this theory that glycosylation, especially core fucosylation, may regulate the function of TNFR. It is also noteworthy that the aberrant expression of FUT8 is commonly found in a variety of tumor cells [13]. Our present results shine a light on the relationship between core fucosylation and membrane receptors in cancers.

About the mechanism, although only a few studies have explored the influence of core fucosylation on membrane receptors in recent years, previously reported findings have important implications for us to solve these problems. For example, normal core fucosylation was demonstrated to be indispensable for the expression of PD-1 on immune cell membranes [41]. PD-1 has long been known to play an important role in how tumors evade the immune system [42]. Likewise, core fucosylation is also required for T-cell receptor transport to their proper place in lipid rafts in T-cell membranes and to transmit T-cell signals properly [43]. In addition, core fucosylation facilitates the binding of TGF-β receptors with their ligands in tumor cells and the phosphorylation of epidermal growth factor receptor in cancer-associated fibroblasts, which could promote the formation of epithelial-mesenchymal transition and tumor microenvironment, respectively [16, 44]. These breakthrough discoveries provide a powerful theoretical foundation for our conclusions and offer hints to some potential mechanisms. In particular, the alteration of FUT8 may play a role in TNFR by affecting their formation and transport, the ability to bind to ligands, and signal transduction, among others.

Although our present findings have illuminated the fairly complete organization and relationships of FUT8, core fucosylation of TNFR, regulation of apoptosis, and the NF-κB2 signaling in OS progression. There is still something should be done in the following studies. Firstly, although our hypothesis has been fully verified in the OS cell and pack of patients, it would be more convincing that this signaling pattern could be widely examined in other OS cell lines and more patients. Secondly, the precise mechanisms involved in this loop especially the cascade between the TNFR and the NF-κB2 signaling remain unclear and need to be further studied. Last but not the least, the present research pointed out the important role of the NF-κB2 signaling pathway in the progression of OS. However, until now, no well-accepted therapeutic inhibitor of this pathway has been applied in clinic. Thus, more studies on FUT8 or other potential antagonist of NF-κB2 signaling should be studied to develop the therapeutic inhibitors in the future.

In general, OS remains one of the fatal malignant tumors affecting the quality of life and health of adolescents, thus, there is an urgent need to improve the curative effects of existing therapies [45]. Although the mechanism underlying alteration of the NF-κB2 signaling pathway following core fucosylation of TNFR should be further studied, our present results show that high expression of FUT8 which result in the suppress of NF-κB2 signaling pathway in OS cells is associated with tumor suppression. Conceivably, increasing the expression of FUT8 or suppress the NF-κB2 signaling pathway in tumor cells of patients with OS may provide a new approach to attenuate the tumor burden of OS in the future.

## Supplementary information

Supplementary files

Reproducibility checklist

## Data Availability

The transcriptomics data generated during this study have been uploaded to NCBI SRA database. The SRA accession number is PRJNA772908. All other data related to this study are available upon request (zhoushumin_zw@126.com).

## References

[1] Isakoff MS, Bielack SS, Meltzer P, Gorlick R (2015). Osteosarcoma: current treatment and a collaborative pathway to success. J Clin Oncol.

[2] Corre I, Verrecchia F, Crenn V, Redini F, Trichet V. The osteosarcoma microenvironment: a complex but targetable ecosystem. Cells. 2020;9. 10.3390/cells9040976 PMC7226971 32326444

[3] Kleinerman E (2016). Maximum benefit of chemotherapy for osteosarcoma achieved—what are the next steps?. Lancet Oncol.

[4] Eichler J (2019). Protein glycosylation. Curr Biol.

[5] Pinho SS, Reis CA (2015). Glycosylation in cancer: mechanisms and clinical implications. Nat Rev Cancer.

[6] Rao X, Duan X, Mao W, Li X, Li Z, Li Q (2015). O-GlcNAcylation of G6PD promotes the pentose phosphate pathway and tumor growth. Nat Commun.

[7] Park DD, Phoomak C, Xu G, Olney LP, Tran KA, Park SS (2020). Metastasis of cholangiocarcinoma is promoted by extended high-mannose glycans. Proc Natl Acad Sci USA.

[8] Croci DO, Cerliani JP, Dalotto-Moreno T, Mendez-Huergo SP, Mascanfroni ID, Dergan-Dylon S (2014). Glycosylation-dependent lectin-receptor interactions preserve angiogenesis in anti-VEGF refractory tumors. Cell.

[9] Wu J, Chen S, Liu H, Zhang Z, Ni Z, Chen J (2018). Tunicamycin specifically aggravates ER stress and overcomes chemoresistance in multidrug-resistant gastric cancer cells by inhibiting N-glycosylation. J Exp Clin Cancer Res.

[10] Dai Y, Cheng Z, Pang Y, Jiao Y, Qian T, Quan L (2020). Prognostic value of the FUT family in acute myeloid leukemia. Cancer Gene Ther.

[11] Keeley TS, Yang S, Lau E The Diverse Contributions of Fucose Linkages in Cancer. Cancers (Basel). 2019;11. 10.3390/cancers11091241 PMC6769556 31450600

[12] Nagae M, Yamaguchi Y, Taniguchi N, Kizuka Y 3D Structure and Function of Glycosyltransferases Involved in N-glycan Maturation. Int J Mol Sci. 2020;21. 10.3390/ijms21020437 PMC7014118 31936666

[13] Bastian K, Scott E, Elliott DJ, Munkley J FUT8 Alpha-(1,6)-Fucosyltransferase in Cancer. Int J Mol Sci. 2021;22. 10.3390/ijms22010455 PMC7795606 33466384

[14] Wang Y, Fukuda T, Isaji T, Lu J, Im S, Hang Q (2015). Loss of alpha1,6-fucosyltransferase inhibits chemical-induced hepatocellular carcinoma and tumorigenesis by down-regulating several cell signaling pathways. FASEB J: Off Publ Federation Am Societies Exp Biol.

[15] Agrawal P, Fontanals-Cirera B, Sokolova E, Jacob S, Vaiana CA, Argibay D (2017). A Systems Biology Approach Identifies FUT8 as a Driver of Melanoma Metastasis. Cancer Cell.

[16] Tu CF, Wu MY, Lin YC, Kannagi R, Yang RB (2017). FUT8 promotes breast cancer cell invasiveness by remodeling TGF-beta receptor core fucosylation. Breast Cancer Res.

[17] Zhao YP, Xu XY, Fang M, Wang H, You Q, Yi CH (2014). Decreased core-fucosylation contributes to malignancy in gastric cancer. PLoS ONE.

[18] Lebourgeois S, Fraisse A, Hennechart-Collette C, Guillier L, Perelle S, Martin-Latil S (2018). Development of a real-time cell analysis (RTCA) method as a fast and accurate method for detecting infectious particles of the adapted strain of hepatitis A virus. Front Cell Infect Microbiol.

[19] Shimosato Y, Kameya T, Nagai K, Hirohashi S, Koide T, Hayashi H (1976). Transplantation of human tumors in nude mice. J Natl Cancer Inst.

[20] Belmokhtar CA, Hillion J, Ségal-Bendirdjian E (2001). Staurosporine induces apoptosis through both caspase-dependent and caspase-independent mechanisms. Oncogene.

[21] Reers M, Smith TW, Chen LB (1991). J-aggregate formation of a carbocyanine as a quantitative fluorescent indicator of membrane potential. Biochemistry.

[22] Sun SC (2017). The non-canonical NF-kappaB pathway in immunity and inflammation. Nat Rev Immunol.

[23] Chen M, Zhao Z, Meng Q, Liang P, Su Z, Wu Y, et al. TRIM14 promotes noncanonical NF‐κB activation by modulating p100/p52 stability via selective autophagy. Advanced Science. 2019;7. 10.1002/advs.201901261 PMC6947505 31921549

[24] Micheau O, Tschopp J (2003). Induction of TNF Receptor I-mediated apoptosis via two sequential signaling complexes. Cell.

[25] Gupta S (2002). Tumor necrosis factor-α-induced apoptosis in T cells from aged humans: a role of TNFR-I and downstream signaling molecules. Exp Gerontol.

[26] Zhang L, Lv B, Shi X, Gao G (2020). High expression of N-acetylgalactosaminyl-transferase 1 (GALNT1) associated with invasion, metastasis, and proliferation in osteosarcoma. Med Sci Monit.

[27] Ajit Varki EE, RD Cummings, JD Esko, P Stanley, GW Hart, M Aebi, et al. Essentials of Glycobiology, 3rd edition. Cold Spring Harbor Laboratory Press: Cold Spring Harbor (NY); 2015–2017.

[28] Deng X, Yi X, Huang D, Liu P, Chen L, Du Y (2020). ROCK2 mediates osteosarcoma progression and TRAIL resistance by modulating O-GlcNAc transferase degradation. Am J Cancer Res.

[29] Baumann S, Hennet T (2016). Collagen accumulation in osteosarcoma cells lacking GLT25D1 collagen galactosyltransferase. J Biol Chem.

[30] Chen CY, Jan YH, Juan YH, Yang CJ, Huang MS, Yu CJ (2013). Fucosyltransferase 8 as a functional regulator of nonsmall cell lung cancer. Proc Natl Acad Sci USA.

[31] Honma R, Kinoshita I, Miyoshi E, Tomaru U, Matsuno Y, Shimizu Y (2015). Expression of fucosyltransferase 8 is associated with an unfavorable clinical outcome in non-small cell lung cancers. Oncology.

[32] Wang X, Chen J, Li QK, Peskoe SB, Zhang B, Choi C (2014). Overexpression of alpha (1,6) fucosyltransferase associated with aggressive prostate cancer. Glycobiology.

[33] Hayden MS, Ghosh S (2014). Regulation of NF-kappaB by TNF family cytokines. Semin Immunol.

[34] Schwarz RT, Datema R (1982). The lipid pathway of protein glycosylation and its inhibitors: the biological significance of protein-bound carbohydrates. Adv Carbohydr Chem Biochem.

[35] Lei Q, Gu H, Li L, Wu T, Xie W, Li M (2020). TNIP1-mediated TNF-alpha/NF-kappaB signalling cascade sustains glioma cell proliferation. J Cell Mol Med.

[36] Ooppachai C, Limtrakul Dejkriengkraikul P, Yodkeeree S. Dicentrine potentiates TNF-alpha-induced apoptosis and suppresses invasion of A549 lung adenocarcinoma cells via modulation of NF-kappaB and AP-1 Activation. Molecules. 2019;24. 10.3390/molecules24224100 PMC6891634 31766230

[37] De Simone V, Franze E, Ronchetti G, Colantoni A, Fantini MC, Di Fusco D (2015). Th17-type cytokines, IL-6 and TNF-alpha synergistically activate STAT3 and NF-kB to promote colorectal cancer cell growth. Oncogene.

[38] Zuo J, Zhao M, Liu B, Han X, Li Y, Wang W (2019). TNFalphamediated upregulation of SOD2 contributes to cell proliferation and cisplatin resistance in esophageal squamous cell carcinoma. Oncol Rep..

[39] de Vreede G, Morrison HA, Houser AM, Boileau RM, Andersen D, Colombani J (2018). A Drosophila Tumor Suppressor Gene Prevents Tonic TNF Signaling through Receptor N-Glycosylation. Dev Cell.

[40] Holdbrooks AT, Britain CM, Bellis SL (2018). ST6Gal-I sialyltransferase promotes tumor necrosis factor (TNF)-mediated cancer cell survival via sialylation of the TNF receptor 1 (TNFR1) death receptor. J Biol Chem.

[41] Okada M, Chikuma S, Kondo T, Hibino S, Machiyama H, Yokosuka T (2017). Blockage of core fucosylation reduces cell-surface expression of PD-1 and promotes anti-tumor immune responses of T cells. Cell Rep..

[42] Syn NL, Teng MWL, Mok TSK, Soo RA (2017). De-novo and acquired resistance to immune checkpoint targeting. Lancet Oncol.

[43] Fujii H, Shinzaki S, Iijima H, Wakamatsu K, Iwamoto C, Sobajima T (2016). Core fucosylation on T cells, required for activation of T-cell receptor signaling and induction of colitis in mice, is increased in patients with inflammatory bowel disease. Gastroenterology.

[44] Li F, Zhao S, Cui Y, Guo T, Qiang J, Xie Q (2020). α1,6-Fucosyltransferase (FUT8) regulates the cancer-promoting capacity of cancer-associated fibroblasts (CAFs) by modifying EGFR core fucosylation (CF) in non-small cell lung cancer (NSCLC). Am J Cancer Res.

[45] Belayneh R, Fourman MS, Bhogal S, Weiss KR (2021). Update on osteosarcoma. Curr Oncol Rep..

