# Supplementary material for: Alpha-(1,6)-fucosyltransferase (FUT8) affects the survival strategy of osteosarcoma by remodeling TNF/NF-κB2 signaling
Source: Cell Death Dis. 2021 Dec 2;12(12):1124. doi: 10.1038/s41419-021-04416-x (PMC8640016; doi:10.1038/s41419-021-04416-x)
Supplement: Supplementary file 1 — Supplementary files [file 41419_2021_4416_MOESM1_ESM.pdf]

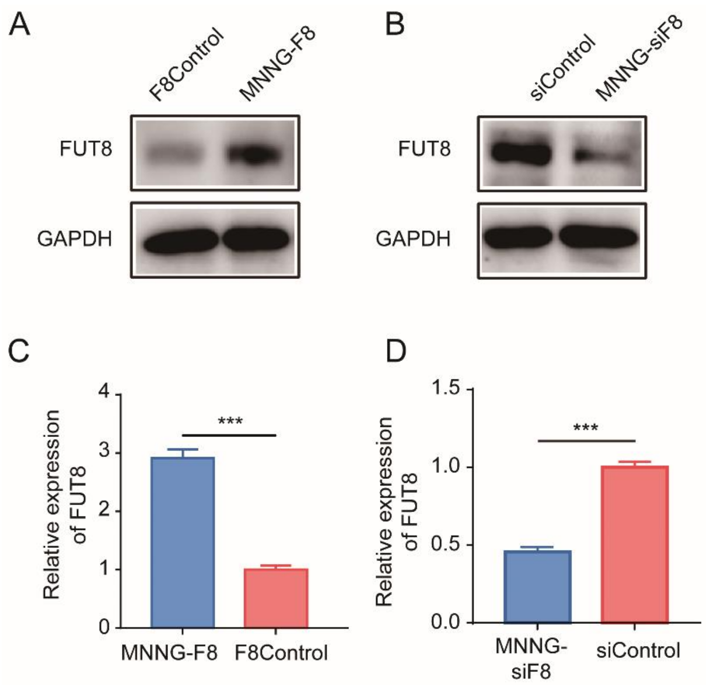

Fig. S1 Successful overexpression and knockdown of FUT8 was verified in transcription and translation level. (A, B) Western blot analysis of FUT8 overexpression and knockdown in MNNG-F8 and MNNG-siF8 cell lines. (C, D) Quantitation of *FUT8* mRNA by qRT-PCR in MNNG-F8 and MNNG-siF8 cell lines, (MNNG-F8, over-expression of FUT8; MNNG-siF8, under-expression of FUT8; control cell lines, F8Control and siControl, respectively). GAPDH in A and B was loading control and levels were calculated relative to GAPDH levels. Values in C and D presented as mean ± SD (N=3); *** $P < 0.001$.

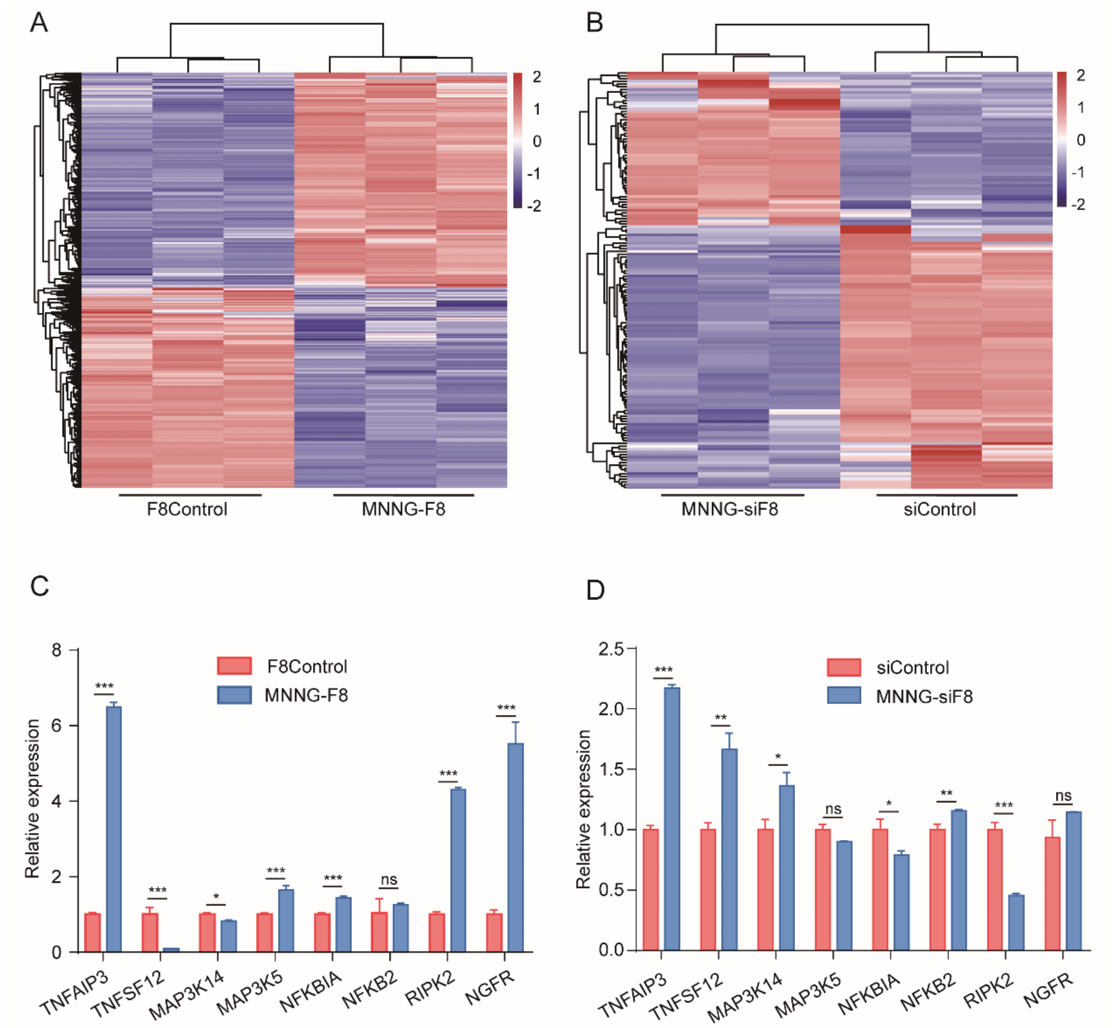

Fig. S2 FUT8 is involved in metastasis in OS cells. (A, B) Mean ± SD (N=4) cell index, a measure of cell migration and cell invasion, for two FUT8-altered OS cell lines (MNNG-F8, MNNG-siF8) and their control cell lines (F8Control, siControl, respectively) over time. RTCA assay confirmed that the "migration ability" was progressively enhanced in the MNNG-siF8 group and slightly but non-significantly reduced in the MNNG-F8 group compared to their control group. (C, D) Similarly, the RTCA assay confirmed that "invasion ability" was enhanced in MNNG-siF8 cells and non-significantly reduced in MNNG-F8 cells compared to control cells.

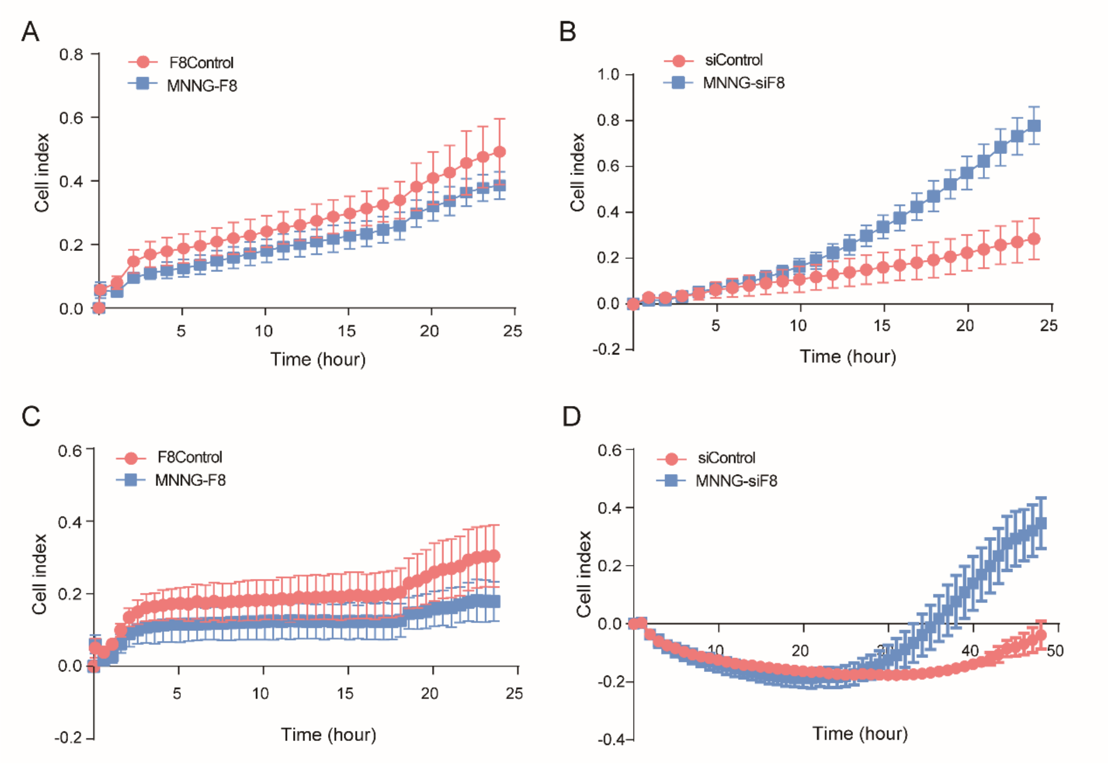

Fig. S3 Transcriptome analysis of FUT8-overexpressing MNNG-F8 and FUT8-under-expressing MNNG-siF8 cells and their control cells. (A-B) The heatmap of DEGs showing there was a different gene expression profile between F8Control cells and MNNG-F8 cells, siControl cells and MNNG-siF8 cells. (C-D) Among the DEGs, the RT-qPCR verified the genes that highly related to the TNF/NFκB pathway in F8Control, MNNG-F8, siControl, and MNNG-siF8 cells, data were shown as mean ± SD (N=3).

Supplementary Table. 1 Information of the vector and sequences of full-length FUT8 and siRNA against FUT8

	Vector Name	Sequences
**siRNA against FUT8**	pLKO.5* shControl	CAACAAGATGAAGAGCACCAA
	pLKO.5 shFUT8-1	GAACTGGTTCAGCGGAGAATA
	pLKO.5 shFUT8-2	TACCCATGCACAGTACAATAA
	pLKO.5 shFUT8-3	CTTTAGATGACATCTACTATT
**Fucosyltransferase 8 (FUT8) (NM_001371533)**	pLVX-EGFP-IRES-puro# FUT8	ATTCGCGCGC GCACGCCGGC GCTGGCCGAG GCTTCCCCGC CTGCGCTCGT TGTCAGAGCC GCTCCGGCGC GTGCGCGCGT TATCTCCGGC CGACCCGAGC AGCCGGTTCC CTCCTCTCCA GGCCCCCTCC CCATCCCACC CCCGCCGCCT GGCCCCAGCC GACCCGTCCC TTCGTCTCCC CGCGGAATGG GGCCGGCACT GCTCAGGGTC GCGCGCCCTG GACCCAGCTC GCTCTCGGTC TCGCGCTGTC AGCGACTGCC CGGCTCGCGC CGCCTCGCGC TCTGCCTCAG TCAGTGGCGC CGAAGGCTCC GTTAAGCGGC GGCGGCGGTT CCTGTTTCCG TTTCTTCCTC TCCGTTCGGT CGGGAGTAGC ATCCTCCACT CAGCCACCCT TCCCACTCCC CCATCGTGGG GCAGCTGCGG CTGAGGGCTG TGGCTTTGGC AGCTGCGACG GGGAGCGGCG GAGACCGCCT CTGCTCCCGC CTGGGGTTGC TGCTTTTGCT CAGAGGACAT CCATGACCCT AATGGTCTTT TTGTTCAAGA TAAAGTGATT TTTTGCCTTT GTTGATTAAC

	TGGACAAATT CAGCATGTAG AGCGCATGAA
	GTACAGGACA ATAAAGCTTC CTACACATAT
	CACCAGGAGG ATCTCTTTGA AAGATTCACT
	GCAGGACTAC CAGAGAGAAT AATTTGTCTG
	AAGCATCATG TGTTGAAACA ACAGAAGTCT
	ATTCACCTGT GCACTAACTA GAAACAGAGT
	TACAATGTTT TCAATTCTTT GAGCTCCAGG
	ACTCCAGGGA AGTGAGTTGA AAATCTGAAA
	ATGCGGCCAT GGACTGGTTC CTGGCGTTGG
	ATTATGCTCA TTCTTTTTGC CTGGGGGACC
	TTGCTGTTTT ATATAGGTGG TCACTTGGTA
	CGAGATAATG ACCATCCTGA TCACTCTAGC
	CGAGAACTGT CCAAGATTCT GGCAAAGCTT
	GAACGCTTAA AACAACAGAA TGAAGACTTG
	AGGCGAATGG CCGAATCTCT CCGGATACCA
	GAAGGCCCTA TTGATCAGGG GCCAGCTATA
	GGAAGAGTAC GCGTTTTAGA AGAGCAGCTT
	GTTAAGGCCA AAGAACAGAT TGAAAATTAC
	AAGAAACAGA CCAGAAATGG TCTGGGGAAG
	GATCATGAAA TCCTGAGGAG GAGGATTGAA
	AATGGAGCTA AAGAGCTCTG GTTTTTCCTA
	CAGAGTGAAT TGAAGAAATT AAAGAACTTA
	GAAGGAAATG AACTCCAAAG ACATGCAGAT
	GAATTTCTTT TGGATTTAGG ACATCATGAA
	AGGTCTATAA TGACGGATCT ATACTACCTC
	AGTCAGACAG ATGGAGCAGG TGATTGGCGG
	GAAAAAGAGG CCAAAGATCT GACAGAACTG
	GTTCAGCGGA GAATAACATA TCTTCAGAAT
	CCCAAGGACT GCAGCAAAGC CAAAAAGCTG

	GTGTGTAATA TCAACAAAGG CTGTGGCTAT
	GGCTGTCAGC TCCATCATGT GGTCTACTGC
	TTCATGATTG CATATGGCAC CCAGCGAACA
	CTCATCTTGG AATCTCAGAA TTGGCGCTAT
	GCTACTGGTG GATGGGAGAC TGTATTTAGG
	CCTGTAAGTG AGACATGCAC AGACAGATCT
	GGCATCTCCA CTGGACACTG GTCAGGTGAA
	GTGAAGGACA AAAATGTTCA AGTGGTCGAG
	CTTCCCATTG TAGACAGTCT TCATCCCCGT
	CCTCCATATT TACCCTTGGC TGTACCAGAA
	GACCTCGCAG ATCGACTTGT ACGAGTGCAT
	GGTGACCCTG CAGTGTGGTG GGTGTCTCAG
	TTTGTCAAAT ACTTGATCCG CCCACAGCCT
	TGGCTAGAAA AAGAAATAGA AGAAGCCACC
	AAGAAGCTTG GCTTCAAACA TCCAGTTATT
	GGAGTCCATG TCAGACGCAC AGACAAAGTG
	GGAACAGAAG CTGCCTTCCA TCCCATTGAA
	GAGTACATGG TGCATGTTGA AGAACATTTT
	CAGCTTCTTG CACGCAGAAT GCAAGTGGAC
	AAAAAAAGAG TGTATTTGGC CACAGATGAC
	CCTTCTTTAT TAAAGGAGGC AAAAACAAAG
	TACCCCAATT ATGAATTTAT TAGTGATAAC
	TCTATTTCCT GGTCAGCTGG ACTGCACAAT
	CGATACACAG AAAATTCACT TCGTGGAGTG
	ATCCTGGATA TACATTTTCT CTCTCAGGCA
	GACTTCCTAG TGTGTACTTT TTCATCCCAG
	GTCTGTCGAG TTGCTTATGA AATTATGCAA
	ACACTACATC CTGATGCCTC TGCAAACTTC
	CATTCTTTAG ATGACATCTA CTATTTTGGG

	GGCCAGAATG CCCACAATCA AATTGCCATT
	TATGCTCACC AACCCCGAAC TGCAGATGAA
	ATTCCCATGG AACCTGGAGA TATCATTGGT
	GTGGCTGGAA ATCATTGGGA TGGCTATTCT
	AAAGGTGTCA ACAGGAAATT GGGAAGGACG
	GGCCTATATC CCTCCTACAA AGTTCGAGAG
	AAGATAGAAA CGGTCAAGTA CCCCACATAT
	CCTGAGGCTG AGAAATAAAG CTCAGATGGA
	AGAGATAAAC GACCAAACTC AGTTCGACCA
	AACTCAGTTC AAACCATTTC AGCCAAACTG
	TAGATGAAGA GGGCTCTGAT CTAACAAAAT
	AAGGTTATAT GAGTAGATAC TCTCAGCACC
	AAGAGCAGCT GGGAACTGAC ATAGGCTTCA
	ATTGGTGGAA TTCCTCTTTA ACAAGGGCTG
	CAATGCCCTC ATACCCATGC ACAGTACAAT
	AATGTACTCA CATATAACAT GCAAACAGGT
	TGTTTTCTAC TTTGCCCCTT TCAGTATGTC
	CCCATAAGAC AAACACTGCC ATATTGTGTA
	ATTTAAGTGA CACAGACATT TTGTGTGAGA
	CTTAAAACAT GGTGCCTATA TCTGAGAGAC
	CTGTGTGAAC TATTGAGAAG ATCGGAACAG
	CTCCTTACTC TGAGGAAGTT GATTCTTATT
	TGATGGTGGT ATTGTGACCA CTGAATTCAC
	TCCAGTCAAC AGATTCAGAA TGAGAATGGA
	CGTTTGGTTT TTTTTTGTTT TTGTTTTTGT
	TTTTTCCTTT ATAAGGTTGT CTGTTTTTTT
	TTTTTTAAAT AATTGCATCA GTTCATTGAC
	CTCATCATTA ATAAGTGAAG AATACATCAG
	AAAATAAAAT ATTCACTCTC CATTAGAAAA

	TTTTGTAAAA CAATGCCATG AACAAATTCT<br>TTAGTACTCA ATGTTTCTGG ACATTCTCTT<br>TGATAACAAA AAATAAATTT TAAAAAGGAA<br>TTTTGTAAAG TTTCTAGAAT<br>TTTATATCATTGGATGATAT GTTGATCAGC<br>CTTATGTGGA AGAACTGTGA TAAAAAGAGG<br>AGCTTTTTAG TTTTTCAGCT TATTTACTTT<br>GTTTTTTTTC CTGTTTCTGA TATAGTAACT<br>AATTCTTAAC TCAGAGACAT TGGTCCATTT<br>TAATACTGAA AACCAATTTT CATTGGTACA<br>CATTACAAAA TTGCTAAGAA CACTGTTTGG<br>GAGCTTTCAT TCTCATATTT TGGACATTGT<br>TTTAATTGAG TGAAATAATC ATAACTCCTT<br>GCTCCCAGAG AAGCTATCAC CTCCATTTCT<br>AAAACCATTT CAGGTTTGTT TGGTAGTCTT<br>TCTTAATGTA TTTATTATCG CTTTTTGTGA<br>GTAGGGTCCT GTTTTATCCA GGAACCAATT<br>TCTGCCCTAG CCTATCATGC CCTGCTTTTG<br>AGAGTACCAG GTATCTTTGG GATTGGGAAG<br>CTGGCTGTTT CAGAAGTATA TGTCTCATAG<br>TGTGCAGAAC TTGGTGACCA AGTGAGAAGC<br>GGGACCAATG GGAGACTCAC AATGGACTGA<br>GTCTTGGGAT TATCTTTTCC AAATTCTTCC<br>ATGTTAGAAT CACTTCAGAA AATAAGACTT<br>TGATGCTTTG TCTCTGAGCA TCATTTTTCT<br>CCTCATAAAA ACACTTCCTT ATTGTATGTA<br>GCCTGCCTCC TACAGAGGCC TGGTAGGTGT<br>TACTGCATTC TAAAAGAAAA ATGTCATCTC<br>TGTAGGAGCG ACTATCAGGC CTAGTGTGAA

	ATATTAGGGA TCCTAGGCAG AAGAGCTATT
	AGTCCTGGCC TTCATATCTT CACCAAATGA
	AAATACTGTA TATAAAATTT CACCACCAAA
	CTTAACTAAA TTCTTTTTCT CAAGTCAAGC
	CTCCCAAAGA AAAAAGAAAT TAACTTCCTA
	CAGTGTCAGC AAGCAATTTT CCATTTAGTT
	TTGGTACAAA TAAAAGTCAT TTGAAACAA

***pLKO_TRC005**

CCCGGGTTGCGCCTTTTCCAAGGCAGCCCTGGGTTTGCGCAGGGACGCGGCTGCTCTG
GGCGTGGTTCCGGGAAACGCAGCGGCGCCGACCCTGGGTCTCGCACATTCTTCACGTC
CGTTCGCAGCGTCACCCGGATCTTCGCCGCTACCCTTGTGGGCCCCCCGGCGACGCTT
CCTGCTCCGCCCCTAAGTCGGGAAGGTTCCTTGCGGTTCGCGGCGTGCCGGACGTGAC
AAACGGAAGCCGCACGTCTCACTAGTACCCTCGCAGACGGACAGCGCCAGGGAGCAA
TGGCAGCGCGCCGACCGCGATGGGCTGTGGCCAATAGCGGCTGCTCAGCAGGGCGCG
CCGAGAGCAGCGGCCGGGAAGGGGCGGTGCGGGAGGCGGGGTGTGGGGCGGTAGTG
TGGGCCCTGTTCCTGCCCGCGCGGTGTTCCGCATTCTGCAAGCCTCCGGAGCGCACGT
CGGCAGTCGGCTCCCTCGTTGACCGAATCACCGACCTCTCTCCCCAGGGGGATCCACC
GGAGCTTACCATGACCGAGTACAAGCCCACGGTGCGCCTCGCCACCCGCGACGACGT
CCCCAGGGCCGTACGCACCCTCGCCGCCGCGTTCGCCGACTACCCCGCCACGCGCCAC
ACCGTCGATCCGGACCGCCACATCGAGCGGGTCACCGAGCTGCAAGAACTCTTCCTCA
CGCGCGTCGGGCTCGACATCGGCAAGGTGTGGGTCGCGGACGACGGCGCCGCCGTGG
CGGTCTGGACCACGCCGGAGAGCGTCGAAGCGGGGGCGGTGTTCGCCGAGATCGGCC
CGCGCATGGCCGAGTTGAGCGGTTCCCGGCTGGCCGCGCAGCAACAGATGGAAGGCC
TCCTGGCGCCGCACCGGCCCAAGGAGCCCGCGTGGTTCCTGGCCACCGTCGGCGTCTC
GCCCGACCACCAGGGCAAGGGTCTGGGCAGCGCCGTCGTGCTCCCCGGAGTGGAGGC
GGCCGAGCGCGCCGGGGTGCCCGCCTTCCTGGAGACCTCCGCGCCCCGCAACCTCCC
CTTCTACGAGCGGCTCGGCTTCACCGTCACCGCCGACGTCGAGGTGCCCGAAGGACC
GCGCACCTGGTGCATGACCCGCAAGCCCGGTGCCTGAACGCGTTAAGTCGACAATCAA
CCTCTGGATTACAAAATTTGTGAAAGATTGACTGGTATTCTTAACTATGTTGCTCCTTTT
ACGCTATGTGGATACGCTGCTTTAATGCCTTTGTATCATGCTATTGCTTCCCGTATGGCTT
TCATTTTCTCCTCCTTGTATAAATCCTGGTTGCTGTCTCTTTATGAGGAGTTGTGGCCCG
TTGTCAGGCAACGTGGCGTGGTGTGCACTGTGTTTGCTGACGCAACCCCCACTGGTTG
GGGCATTGCCACCACCTGTCAGCTCCTTTCCGGGACTTTCGCTTTCCCCCTCCCTATTG
CCACGGCGGAACTCATCGCCGCCTGCCTTGCCCGCTGCTGGACAGGGGCTCGGCTGTT
GGGCACTGACAATTCCGTGGTGTTGTCGGGGAAATCATCGTCCTTTCCTTGGCTGCTCG

CCTGTGTTGCCACCTGGATTCTGCGCGGGACGTCCTTCTGCTACGTCCCTTCGGCCCTC
AATCCAGCGGACCTTCCTTCCCGCGGCCTGCTGCCGGCTCTGCGGCCTCTTCCGCGTCT
TCGCCTTCGCCCTCAGACGAGTCGGATCTCCCTTTGGGCCGCCTCCCCGCGTCGACTTT
AAGACCAATGACTTACAAGGCAGCTGTAGATCTTAGCCACTTTTTAAAAGAAAAGGGG
GGACTGGAAGGGCTAATTCACTCCCAACGAAGACAAGATCTGCTTTTTGCTTGTACTG
GGTCTCTCTGGTTAGACCAGATCTGAGCCTGGGAGCTCTCTGGCTAACTAGGGAACCC
ACTGCTTAAGCCTCAATAAAGCTTGCCTTGAGTGCTTCAAGTAGTGTGTGCCCGTCTGT
TGTGTGACTCTGGTAACTAGAGATCCCTCAGACCCTTTTAGTCAGTGTGGAAAATCTCT
AGCAGTACGTATAGTAGTTCATGTCATCTTATTATTCAGTATTTATAACTTGCAAAGAAAT
GAATATCAGAGAGTGAGAGGAACTTGTTTATTGCAGCTTATAATGGTTACAAATAAAGC
AATAGCATCACAAATTTCACAAATAAAGCATTTTTTTCACTGCATTCTAGTTGTGGTTTG
TCCAAACTCATCAATGTATCTTATCATGTCTGGCTCTAGCTATCCCGCCCCTAACTCCGC
CCATCCCGCCCCTAACTCCGCCCAGTTCCGCCCATTCTCCGCCCCATGGCTGACTAATT
TTTTTTATTTATGCAGAGGCCGAGGCCGCCTCGGCCTCTGAGCTATTCCAGAAGTAGTG
AGGAGGCTTTTTTGGAGGCCTAGGGACGTACCCAATTCGCCCTATAGTGAGTCGTATTA
CGCGCGCTCACTGGCCGTCGTTTTACAACGTCGTGACTGGGAAAACCCTGGCGTTACC
CAACTTAATCGCCTTGCAGCACATCCCCCTTTCGCCAGCTGGCGTAATAGCGAAGAGG
CCCGCACCGATCGCCCTTCCCAACAGTTGCGCAGCCTGAATGGCGAATGGGACGCGCC
CTGTAGCGGCGCATTAAGCGCGGCGGGTGTGGTGGTTACGCGCAGCGTGACCGCTACA
CTTGCCAGCGCCCTAGCGCCCGCTCCTTTCGCTTTCTTCCCTTCCTTTCTCGCCACGTTC
GCCGGCTTTCCCCGTCAAGCTCTAAATCGGGGGCTCCCTTTAGGGTTCCGATTTAGTGC
TTTACGGCACCTCGACCCCAAAAAACTTGATTAGGGTGATGGTTCACGTAGTGGGCCA
TCGCCCTGATAGACGGTTTTTCGCCCTTTGACGTTGGAGTCCACGTTCTTTAATAGTGG
ACTCTTGTTCCAAACTGGAACAACACTCAACCCTATCTCGGTCTATTCTTTTGATTTATA
AGGGATTTTGCCGATTTCGGCCTATTGGTTAAAAAATGAGCTGATTTAACAAAAATTTA
ACGCGAATTTTAACAAAATATTAACGCTTACAATTTAGGTGGCACTTTTCGGGGAAATG
TGCGCGGAACCCCTATTTGTTTATTTTTCTAAATACATTCAAATATGTATCCGCTCATGAG
ACAATAACCCTGATAAATGCTTCAATAATATTGAAAAAGGAAGAGTATGAGTATTCAAC
ATTTCCGTGTCGCCCTTATTCCCTTTTTTGCGGCATTTTGCCTTCCTGTTTTTGCTCACCC
AGAAACGCTGGTGAAAGTAAAAGATGCTGAAGATCAGTTGGGTGCACGAGTGGGTTA
CATCGAACTGGATCTCAACAGCGGTAAGATCCTTGAGAGTTTTCGCCCCGAAGAACGT
TTTCCAATGATGAGCACTTTTAAAGTTCTGCTATGTGGCGCGGTATTATCCCGTATTGAC
GCCGGGCAAGAGCAACTCGGTCGCCGCATACACTATTCTCAGAATGACTTGGTTGAGT
ACTCACCAGTCACAGAAAAGCATCTTACGGATGGCATGACAGTAAGAGAATTATGCAG
TGCTGCCATAACCATGAGTGATAACACTGCGGCCAACTTACTTCTGACAACGATCGGA
GGACCGAAGGAGCTAACCGCTTTTTTGCACAACATGGGGGATCATGTAACTCGCCTTG
ATCGTTGGGAACCGGAGCTGAATGAAGCCATACCAAACGACGAGCGTGACACCACGA
TGCCTGTAGCAATGGCAACAACGTTGCGCAAACTATTAACTGGCGAACTACTTACTCTA

GCTTCCCGGCAACAATTAATAGACTGGATGGAGGCGGATAAAGTTGCAGGACCACTTC
TGCGCTCGGCCCTTCCGGCTGGCTGGTTTATTGCTGATAAATCTGGAGCCGGTGAGCGT
GGGTCTCGCGGTATCATTGCAGCACTGGGGCCAGATGGTAAGCCCTCCCGTATCGTAGT
TATCTACACGACGGGGAGTCAGGCAACTATGGATGAACGAAATAGACAGATCGCTGAG
ATAGGTGCCTCACTGATTAAGCATTGGTAACTGTCAGACCAAGTTTACTCATATATACTT
TAGATTGATTTAAAACTTCATTTTTAATTTAAAAGGATCTAGGTGAAGATCCTTTTTGAT
AATCTCATGACCAAAATCCCTTAACGTGAGTTTTCGTTCCACTGAGCGTCAGACCCCGT
AGAAAAGATCAAAGGATCTTCTTGAGATCCTTTTTTTCTGCGCGTAATCTGCTGCTTGC
AAACAAAAAAACCACCGCTACCAGCGGTGGTTTGTTTGCCGGATCAAGAGCTACCAA
CTCTTTTTCCGAAGGTAACTGGCTTCAGCAGAGCGCAGATACCAAATACTGTTCTTCTA
GTGTAGCCGTAGTTAGGCCACCACTTCAAGAACTCTGTAGCACCGCCTACATACCTCGC
TCTGCTAATCCTGTTACCAGTGGCTGCTGCCAGTGGCGATAAGTCGTGTCTTACCGGGT
TGGACTCAAGACGATAGTTACCGGATAAGGCGCAGCGGTCGGGCTGAACGGGGGGTT
CGTGCACACAGCCCAGCTTGGAGCGAACGACCTACACCGAACTGAGATACCTACAGC
GTGAGCTATGAGAAAGCGCCACGCTTCCCGAAGGGAGAAAGGCGGACAGGTATCCGG
TAAGCGGCAGGGTCGGAACAGGAGAGCGCACGAGGGAGCTTCCAGGGGGAAACGCC
TGGTATCTTTATAGTCCTGTCGGGTTTCGCCACCTCTGACTTGAGCGTCGATTTTTGTGA
TGCTCGTCAGGGGGGCGGAGCCTATGGAAAAACGCCAGCAACGCGGCCTTTTTACGG
TTCCTGGCCTTTTGCTGGCCTTTTGCTCACATGTTCTTTCCTGCGTTATCCCCTGATTCT
GTGGATAACCGTATTACCGCCTTTGAGTGAGCTGATACCGCTCGCCGCAGCCGAACGA
CCGAGCGCAGCGAGTCAGTGAGCGAGGAAGCGGAAGAGCGCCCAATACGCAAACCG
CCTCTCCCCGCGCGTTGGCCGATTCATTAATGCAGCTGGCACGACAGGTTTCCCGACTG
GAAAGCGGGCAGTGAGCGCAACGCAATTAATGTGAGTTAGCTCACTCATTAGGCACCC
CAGGCTTTACACTTTATGCTTCCGGCTCGTATGTTGTGTGGAATTGTGAGCGGATAACA
ATTTCACACAGGAAACAGCTATGACCATGATTACGCCAAGCGCGCAATTAACCCTCACT
AAAGGGAACAAAAGCTGGAGCTGCAAGCTTAATGTAGTCTTATGCAATACTCTTGTAG
TCTTGCAACATGGTAACGATGAGTTAGCAACATGCCTTACAAGGAGAGAAAAAGCACC
GTGCATGCCGATTGGTGGAAGTAAGGTGGTACGATCGTGCCTTATTAGGAAGGCAACA
GACGGGTCTGACATGGATTGGACGAACCACTGAATTGCCGCATTGCAGAGATATTGTAT
TTAAGTGCCTAGCTCGATACATAAACGGGTCTCTCTGGTTAGACCAGATCTGAGCCTGG
GAGCTCTCTGGCTAACTAGGGAACCCACTGCTTAAGCCTCAATAAAGCTTGCCTTGAG
TGCTTCAAGTAGTGTGTGCCCGTCTGTTGTGTGACTCTGGTAACTAGAGATCCCTCAGA
CCCTTTTAGTCAGTGTGGAAAATCTCTAGCAGTGGCGCCCGAACAGGGACTTGAAAGC
GAAAGGGAAACCAGAGGAGCTCTCTCGACGCAGGACTCGGCTTGCTGAAGCGCGCAC
GGCAAGAGGCGAGGGGCGGCGACTGGTGAGTACGCCAAAAATTTTGACTAGCGGAGG
CTAGAAGGAGAGAGATGGGTGCGAGAGCGTCAGTATTAAGCGGGGGAGAATTAGATC
GCGATGGGAAAAAATTCGGTTAAGGCCAGGGGGAAAGAAAAAATATAAATTAAAACAT
ATAGTATGGGCAAGCAGGGAGCTAGAACGATTCGCAGTTAATCCTGGCCTGTTAGAAA

CATCAGAAGGCTGTAGACAAATACTGGGACAGCTACAACCATCCCTTCAGACAGGATC
AGAAGAACTTAGATCATTATATAATACAGTAGCAACCCTCTATTGTGTGCATCAAAGGAT
AGAGATAAAAGACACCAAGGAAGCTTTAGACAAGATAGAGGAAGAGCAAAACAAAA
GTAAGACCACCGCACAGCAAGCGGCCGCTGATCTTCAGACCTGGAGGAGGAGATATG
AGGGACAATTGGAGAAGTGAATTATATAAATATAAAGTAGTAAAAATTGAACCATTAGG
AGTAGCACCCACCAAGGCAAAGAGAAGAGTGGTGCAGAGAGAAAAAAGAGCAGTGG
GAATAGGAGCTTTGTTCCTTGGGTTCTTGGGAGCAGCAGGAAGCACTATGGGCGCAGC
GTCAATGACGCTGACGGTACAGGCCAGACAATTATTGTCTGGTATAGTGCAGCAGCAG
AACAATTTGCTGAGGGCTATTGAGGCGCAACAGCATCTGTTGCAACTCACAGTCTGGG
GCATCAAGCAGCTCCAGGCAAGAATCCTGGCTGTGGAAAGATACCTAAAGGATCAACA
GCTCCTGGGGATTTGGGGTTGCTCTGGAAAACTCATTTGCACCACTGCTGTGCCTTGG
AATGCTAGTTGGAGTAATAAATCTCTGGAACAGATTTGGAATCACACGACCTGGATGGA
GTGGGACAGAGAAATTAACAATTACACAAGCTTAATACACTCCTTAATTGAAGAATCGC
AAAACCAGCAAGAAAAGAATGAACAAGAATTATTGGAATTAGATAAATGGGCAAGTTT
GTGGAATTGGTTTAACATAACAAATTGGCTGTGGTATATAAAATTATTCATAATGATAGT
AGGAGGCTTGGTAGGTTTAAGAATAGTTTTTGCTGTACTTTCTATAGTGAATAGAGTTA
GGCAGGGATATTCACCATTATCGTTTCAGACCCACCTCCCAACCCCGAGGGGACCCAG
AGAGGGCCTATTTCCCATGATTCCTTCATATTTGCATATACGATACAAGGCTGTTAGAGA
GATAATTAGAATTAATTTGACTGTAAACACAAAGATATTAGTACAAAATACGTGACGTA
GAAAGTAATAATTTCTTGGGTAGTTTGCAGTTTTAAAATTATGTTTTAAAATGGACTATC
ATATGCTTACCGTAACTTGAAAGTATTTCGATTTCTTGGCTTTATATATCTTGTGGAAAGG
ACGAGGTACCGGTGTGTTGTAAATGAGCACACAAAATACACATGCTAAAATATTATATT
CTATGACCTTTATAAAATCAACCAAAATCTTCTTTTTAATAACTTTAGTATCAATAATTAG
AATTTTTATGTTCCTTTTTGCAAACTTTTAATAAAAATGAGCAAAATAAAAAAACGCTA
GTTTTAGTAACTCGCGTTGTTTTCTTCACCTTTAATAATAGCTACTCCACCACTTGTTCC
TAAGCGGTCAGCTCCTGCTTCAATCATTTTTTGAGCATCTTCAAATGTTCTAACTCCACC
AGCTGCTTTAACTAAAGCATTGTCTTTAACAACTGACTTCATTAGTTTAACATCTTCAAA
TGTTGCACCTGATTTTGAAAATCCTGTTGATGTTTTAACAAATTCTAATCCAGCTTCAAC
AGCTATTTCACAAGCTTTCATGATTTCTTCTTTTGTTAATAAACAATTTTCCATAATACAT
TTAACAACATGTGATCCAGCTGCTTTTTTTACAGCTTTCATGTCTTCTAAAACTAATTCA
TAATTTTTGTCTTTTAATGCACCAATATTTAATACCATATCAATTTCTGTTGCACCATCTTT
AATTGCTTCAGAAACTTCGAATGCTTTTGTAGCTGTTGTGCATGCACCTAGAGGAAAAC
CTACAACATTTGTTATTCCTACATTTGTGCCTTTTAATAATTCTTTACAATAGCTTGTTCA
ATATGAATTAACACAAACTGTTGCAAAATCAAATTCAATTGCTTCATCACATAATTGTTT
AATTTCAGCTTTCGTAGCATCTTGTTTTAATAATGTGTGATCTATATATTTGTTTAGTTTCA
TTTTTTCTCCTATATATTCATTTTTAATTTTAATTCTTTAATAATTTCGTCTACTTTAACTTT
AGCGTTTTGAACAGATTCACCAACACCTATAAAATAAATTTTTAGTTTAGGTTCAGTTCC
ACTTGGGCGAACAGCAAATCATGACTTATCTTCTAAATAAAATTTTAGTAAGTCTTGTCC

TGGCATATTATACATTCCATCGATGTAGTCTTCAACATTAACAACTTTAAGTCCAGCAAT
TTGAGTTAAGGGTGTTGCTCTCAATGATTTCATTAATGGTTCAATTTTTAATTTCTTTTCT
TCTGGTTTAAAATTCAAGTTTAAAGTGAAAGTGTAATATGCACCCATTTCTTTAAATAAA
TCTTCTAAATAGTCTACTAATGTTTTATTTTGTTTTTTATAAAATCAAGCAGCCTCTGCTA
TTAATATAGAAGCTTGTATTCCATCTTTATCTCTAGCTGAGTCATCAAT

TACATATCCATAACTTTCTTCATAAGCAAAAACAAAATTTAATCCGTTATCTTCTTCTTTA
GCAATTTCTCTACCCATTCATTTAAATCCAGTTAAAGTTTTTACAATATTAACTCCATATT
TTTCATGAGCGATTCTATCACCCAAATCACTTGTTACAAAACTTGAATATAGAGCCGGAT
TTTTTGGAATGCTATTTAAGCGTTTTAGATTTGATAATTTCAATCAATTAAAATTGGTCC
TGTTTGATTTCCATCTAATCTTACAAAATGACCATCATGTTTTATTGCCATTCCAAATCTG
TCAGCATCTGGGTCATTCATAATAATAATATCTGCATCATGTTTAATACCATATTCAAGCG
GTATTTTTCATGCAGGATCAAATTCTGGATTTGGATTTACAACATTTTTAAATGTTTCATC
TTCAAATGCATGCTCTTCAACCTCAATAACGTTATATCCTGATTCACGTAATATTTTTGGG
GTAAATTTAGTTCCTGTTCCATTAACTGCGCTAAAAATAATTTTTAAATCTTTTTTAGCTT
CTTGCTCTTTTTTGTAGAATTCTAGATCTTGAGACAAATGGCAGTATTCATCCACAATTT
TAAAAGAAAAGGGGGGATTGGGGGGTACAGTGCAGGGGAAAGAATAGTAGACATAAT
AGCAACAGACATACAAACTAAAGAATTACAAAAACAAATTACAAAAATTCAAAATTTT
CGGGTTTATTACAGGGACAGCAGAGATCCACTTTGGCGCCGGCTCGAGGGGG

#pLVX-EGFP-IRES-puro Sequences

TGGAAGGGCTAATTCACTCCCAAAGAAGACAAGATATCCTTGATCTGTGGATCTACCAC
ACACAAGGCTACTTCCCTGATTAGCAGAACTACACACCAGGGCCAGGGGTCAGATATC
CACTGACCTTTGGATGGTGCTACAAGCTAGTACCAGTTGAGCCAGATAAGGTAGAAGA
GGCCAATAAAGGAGAGAACACCAGCTTGTTACACCCTGTGAGCCTGCATGGGATGGAT
GACCCGGAGAGAGAAGTGTTAGAGTGGAGGTTTGACAGCCGCCTAGCATTTCATCACG
TGGCCCGAGAGCTGCATCCGGAGTACTTCAAGAACTGCTGATATCGAGCTTGCTACAA
GGGACTTTCCGCTGGGGACTTTCCAGGGAGGCGTGGCCTGGGCGGGACTGGGGAGTG
GCGAGCCCTCAGATCCTGCATATAAGCAGCTGCTTTTTGCCTGTACTGGGTCTCTCTGG
TTAGACCAGATCTGAGCCTGGGAGCTCTCTGGCTAACTAGGGAACCCACTGCTTAAGC
CTCAATAAAGCTTGCCTTGAGTGCTTCAAGTAGTGTGTGCCCGTCTGTTGTGTGACTCT
GGTAACTAGAGATCCCTCAGACCCTTTTAGTCAGTGTGGAAAATCTCTAGCAGTGGCG
CCCGAACAGGGACTTGAAAGCGAAAGGGAAACCAGAGGAGCTCTCTCGACGCAGGA
CTCGGCTTGCTGAAGCGCGCACGGCAAGAGGCGAGGGGCGGCGACTGGTGAGTACGC
CAAAAATTTTGACTAGCGGAGGCTAGAAGGAGAGAGATGGGTGCGAGAGCGTCAGTA
TTAAGCGGGGGAGAATTAGATCGCGATGGGAAAAAATTCGGTTAAGGCCAGGGGGAA
AGAAAAAATATAAATTAAAACATATAGTATGGGCAAGCAGGGAGCTAGAACGATTCGC
AGTTAATCCTGGCCTGTTAGAAACATCAGAAGGCTGTAGACAAATACTGGGACAGCTA
CAACCATCCCTTCAGACAGGATCAGAAGAACTTAGATCATTATATAATACAGTAGCAAC

CCTCTATTGTGTGCATCAAAGGATAGAGATAAAAGACACCAAGGAAGCTTTAGACAAG
ATAGAGGAAGAGCAAAACAAAAGTAAGACCACCGCACAGCAAGCGGCCGGCCGCTG
ATCTTCAGACCTGGAGGAGGAGATATGAGGGACAATTGGAGAAGTGAATTATATAAATA
TAAAGTAGTAAAAATTGAACCATTAGGAGTAGCACCCACCAAGGCAAAGAGAAGAGT
GGTGCAGAGAGAAAAAAGAGCAGTGGGAATAGGAGCTTTGTTCCTTGGGTTCTTGGG
AGCAGCAGGAAGCACTATGGGCGCAGCGTCAATGACGCTGACGGTACAGGCCAGACA
ATTATTGTCTGGTATAGTGCAGCAGCAGAACAATTTGCTGAGGGCTATTGAGGCGCAAC
AGCATCTGTTGCAACTCACAGTCTGGGGCATCAAGCAGCTCCAGGCAAGAATCCTGGC
TGTGGAAAGATACCTAAAGGATCAACAGCTCCTGGGGATTTGGGGTTGCTCTGGAAAA
CTCATTTGCACCACTGCTGTGCCTTGGAATGCTAGTTGGAGTAATAAATCTCTGGAACA
GATTTGGAATCACACGACCTGGATGGAGTGGGACAGAGAAATTAACAATTACACAAGC
TTAATACACTCCTTAATTGAAGAATCGCAAAACCAGCAAGAAAAGAATGAACAAGAAT
TATTGGAATTAGATAAATGGGCAAGTTTGTGGAATTGGTTTAACATAACAAATTGGCTG
TGGTATATAAAATTATTCATAATGATAGTAGGAGGCTTGGTAGGTTTAAGAATAGTTTTT
GCTGTACTTTCTATAGTGAATAGAGTTAGGCAGGGATATTCACCATTATCGTTTCAGACC
CACCTCCCAACCCCGAGGGGACCCGACAGGCCCGAAGGAATAGAAGAAGAAGGTGG
AGAGAGAGACAGAGACAGATCCATTCGATTAGTGAACGGATCTCGACGGTATCGCCTT
TAAAAGAAAAGGGGGGATTGGGGGGTACAGTGCAGGGGAAAGAATAGTAGACATAAT
AGCAACAGACATACAAACTAAAGAATTACAAAAACAAATTACAAAAATTCAAAATTTT
CGGGTTTATTACAGGGACAGCAGAGATCCAGTTTATCGATAAGCTTGGGAGTTCCGCGT
TACATAACTTACGGTAAATGGCCCGCCTGGCTGACCGCCCAACGACCCCCGCCCATTG
ACGTCAATAATGACGTATGTTCCCATAGTAACGCCAATAGGGACTTTCCATTGACGTCA
ATGGGTGGAGTATTTACGGTAAACTGCCCACTTGGCAGTACATCAAGTGTATCATATGC
CAAGTACGCCCCCTATTGACGTCAATGACGGTAAATGGCCCGCCTGGCATTATGCCCAG
TACATGACCTTATGGGACTTTCCTACTTGGCAGTACATCTACGTATTAGTCATCGCTATTA
CCATGGTGATGCGGTTTTGGCAGTACATCAATGGGCGTGGATAGCGGTTTGACTCACGG
GGATTTCCAAGTCTCCACCCCATTGACGTCAATGGGAGTTTGTTTTGGCACCAAAATCA
ACGGGACTTTCCAAAATGTCGTAACAACTCCGCCCCATTGACGCAAATGGGCGGTAGG
CGTGTACGGTGGGAGGTCTATATAAGCAGAGCTCGTTTAGTGAACCGTCAGATCGCCTG
GAGACGCCATCCACGCTGTTTTGACCTCCATAGAAGACACCGACTCTACTAGAGGATC
TATTTCCGGTGAATTCCTCGAGACTAGATCAACAAGTTTGTACAAAAAAGCAGGCTCC
GCGGCCGCCCCCTTCACCATGGTGAGCAAGGGCGAGGAGCTGTTCACCGGGGTGGTG
CCCATCCTGGTCGAGCTGGACGGCGACGTAAACGGCCACAAGTTCAGCGTGTCCGGC
GAGGGCGAGGGCGATGCCACCTACGGCAAGCTGACCCTGAAGTTCATCTGCACCACC
GGCAAGCTGCCCGTGCCCTGGCCCACCCTCGTGACCACCCTGACCTACGGCGTGCAGT
GCTTCAGCCGCTACCCCGACCACATGAAGCAGCACGACTTCTTCAAGTCCGCCATGCC
CGAAGGCTACGTCCAGGAGCGCACCATCTTCTTCAAGGACGACGGCAACTACAAGAC
CCGCGCCGAGGTGAAGTTCGAGGGCGACACCCTGGTGAACCGCATCGAGCTGAAGGG

CATCGACTTCAAGGAGGACGGCAACATCCTGGGGCACAAGCTGGAGTACAACTACAA
CAGCCACAACGTCTATATCATGGCCGACAAGCAGAAGAACGGCATCAAGGTGAACTTC
AAGATCCGCCACAACATCGAGGACGGCAGCGTGCAGCTCGCCGACCACTACCAGCAG
AACACCCCCATCGGCGACGGCCCCGTGCTGCTGCCCGACAACCACTACCTGAGCACCC
AGTCCGCCCTGAGCAAAGACCCCAACGAGAAGCGCGATCACATGGTCCTGCTGGAGT
TCGTGACCGCCGCCGGGATCACTCTCGGCATGGACGAGCTGTACAAGTAAAAGGGTG
GGCGCGCCGACCCAGCTTTCTTGTACAAAGTGGTTGATCTAGTTCTAGAGCGGCCGCG
GATCCCGCCCCTCTCCCTCCCCCCCCCCTAACGTTACTGGCCGAAGCCGCTTGGAATAA
GGCCGGTGTGCGTTTGTCTATATGTTATTTTCCACCATATTGCCGTCTTTTGGCAATGTG
AGGGCCCGGAAACCTGGCCCTGTCTTCTTGACGAGCATTCCTAGGGGTCTTTCCCCTCT
CGCCAAAGGAATGCAAGGTCTGTTGAATGTCGTGAAGGAAGCAGTTCCTCTGGAAGC
TTCTTGAAGACAAACAACGTCTGTAGCGACCCTTTGCAGGCAGCGGAACCCCCCACCT
GGCGACAGGTGCCTCTGCGGCCAAAAGCCACGTGTATAAGATACACCTGCAAAGGCG
GCACAACCCCAGTGCCACGTTGTGAGTTGGATAGTTGTGGAAAGAGTCAAATGGCTCT
CCTCAAGCGTATTCAACAAGGGGCTGAAGGATGCCCAGAAGGTACCCCATTGTATGGG
ATCTGATCTGGGGCCTCGGTGCACATGCTTTACATGTGTTTAGTCGAGGTTAAAAAAAC
GTCTAGGCCCCCCGAACCACGGGGACGTGGTTTTCCTTTGAAAAACACGATGATAAGC
TTGCCACAACCCACAAGGAGACGACCTTCCATGACCGAGTACAAGCCCACGGTGCGC
CTCGCCACCCGCGACGACGTCCCCCGGGCCGTACGCACCCTCGCCGCCGCGTTCGCCG
ACTACCCCGCCACGCGCCACACCGTCGACCCGGACCGCCACATCGAGCGGGTCACCG
AGCTGCAAGAACTCTTCCTCACGCGCGTCGGGCTCGACATCGGCAAGGTGTGGGTCG
CGGACGACGGCGCCGCGGTGGCGGTCTGGACCACGCCGGAGAGCGTCGAAGCGGGG
GCGGTGTTCGCCGAGATCGGCCCGCGCATGGCCGAGTTGAGCGGTTCCCGGCTGGCCG
CGCAGCAACAGATGGAAGGCCTCCTGGCGCCGCACCGGCCCAAGGAGCCCGCGTGGT
TCCTGGCCACCGTCGGCGTCTCGCCCGACCACCAGGGCAAGGGTCTGGGCAGCGCCG
TCGTGCTCCCCGGAGTGGAGGCGGCCGAGCGCGCCGGGGTGCCCGCCTTCCTGGAGA
CCTCCGCGCCCCGCAACCTCCCCTTCTACGAGCGGCTCGGCTTCACCGTCACCGCCGA
CGTCGAGGTGCCCGAAGGACCGCGCACCTGGTGCATGACCCGCAAGCCCGGTGCCTA
GACGCGTCTGGAACAATCAACCTCTGGATTACAAAATTTGTGAAAGATTGACTGGTATT
CTTAACTATGTTGCTCCTTTTACGCTATGTGGATACGCTGCTTTAATGCCTTTGTATCATG
CTATTGCTTCCCGTATGGCTTTCATTTTCTCCTCCTTGTATAAATCCTGGTTGCTGTCTCT
TTATGAGGAGTTGTGGCCCGTTGTCAGGCAACGTGGCGTGGTGTGCACTGTGTTTGCT
GACGCAACCCCCACTGGTTGGGGCATTGCCACCACCTGTCAGCTCCTTTCCGGGACTT
TCGCTTTCCCCCTCCCTATTGCCACGGCGGAACTCATCGCCGCCTGCCTTGCCCGCTGC
TGGACAGGGGCTCGGCTGTTGGGCACTGACAATTCCGTGGTGTTGTCGGGGAAGCTG
ACGTCCTTTCCATGGCTGCTCGCCTGTGTTGCCACCTGGATTCTGCGCGGGACGTCCTT
CTGCTACGTCCCTTCGGCCCTCAATCCAGCGGACCTTCCTTCCCGCGGCCTGCTGCCGG
CTCTGCGGCCTCTTCCGCGTCTTCGCCTTCGCCCTCAGACGAGTCGGATCTCCCTTTGG

GCCGCCTCCCCGCCTGGAATTAATTCTGCAGTCGAGACCTAGAAAAACATGGAGCAAT
CACAAGTAGCAATACAGCAGCTACCAATGCTGATTGTGCCTGGCTAGAAGCACAAGAG
GAGGAGGAGGTGAGTTTTCCAGTCACACCTCAGGTACCTTTAAGACCAATGACTTACA
AGGCAGCTGTAGATCTTAGCCACTTTTTAAAAGAAAAGAGGGGACTGGAAGGGCTAAT
TCACTCCCAACGAAGACAAGATATCCTTGATCTGTGGATCTACCACACACAAGGCTACT
TCCCTGATTAGCAGAACTACACACCAGGGCCAGGGGTCAGATATCCACTGACCTTTGG
ATGGTGCTACAAGCTAGTACCAGTTGAGCCAGATAAGGTAGAAGAGGCCAATAAAGGA
GAGAACACCAGCTTGTTACACCCTGTGAGCCTGCATGGGATGGATGACCCGGAGAGA
GAAGTGTTAGAGTGGAGGTTTGACAGCCGCCTAGCATTTCATCACGTGGCCCGAGAGC
TGCATCCGGAGTACTTCAAGAACTGCTGATATCGAGCTTGCTACAAGGGACTTTCCGCT
GGGGACTTTCCAGGGAGGCGTGGCCTGGGCGGGACTGGGGAGTGGCGAGCCCTCAG
ATCCTGCATATAAGCAGCTGCTTTTTGCCTGTACTGGGTCTCTCTGGTTAGACCAGATCT
GAGCCTGGGAGCTCTCTGGCTAACTAGGGAACCCACTGCTTAAGCCTCAATAAAGCTT
GCCTTGAGTGCTTCAAGTAGTGTGTGCCCGTCTGTTGTGTGACTCTGGTAACTAGAGAT
CCCTCAGACCCTTTTAGTCAGTGTGGAAAATCTCTAGCAGTAGTAGTTCATGTCATCTT
ATTATTCAGTATTTATAACTTGCAAAGAAATGAATATCAGAGAGTGAGAGGCCTTGACA
TTGCTAGCGTTTACCGTCGACCTCTAGCTAGAGCTTGGCGTAATCATGGTCATAGCTGTT
TCCTGTGTGAAATTGTTATCCGCTCACAATTCCACACAACATACGAGCCGGAAGCATAA
AGTGTAAAGCCTGGGGTGCCTAATGAGTGAGCTAACTCACATTAATTGCGTTGCGCTCA
CTGCCCGCTTTCCAGTCGGGAAACCTGTCGTGCCAGCTGCATTAATGAATCGGCCAAC
GCGCGGGGAGAGGCGGTTTGCGTATTGGGCGCTCTTCCGCTTCCTCGCTCACTGACTC
GCTGCGCTCGGTCGTTCGGCTGCGGCGAGCGGTATCAGCTCACTCAAAGGCGGTAATA
CGGTTATCCACAGAATCAGGGGATAACGCAGGAAAGAACATGTGAGCAAAAGGCCAG
CAAAAGGCCAGGAACCGTAAAAAGGCCGCGTTGCTGGCGTTTTTCCATAGGCTCCGCC
CCCCTGACGAGCATCACAAAAATCGACGCTCAAGTCAGAGGTGGCGAAACCCGACAG
GACTATAAAGATACCAGGCGTTTCCCCCTGGAAGCTCCCTCGTGCGCTCTCCTGTTCCG
ACCCTGCCGCTTACCGGATACCTGTCCGCCTTTCTCCCTTCGGGAAGCGTGGCGCTTTC
TCATAGCTCACGCTGTAGGTATCTCAGTTCGGTGTAGGTCGTTCGCTCCAAGCTGGGCT
GTGTGCACGAACCCCCCGTTCAGCCCGACCGCTGCGCCTTATCCGGTAACTATCGTCTT
GAGTCCAACCCGGTAAGACACGACTTATCGCCACTGGCAGCAGCCACTGGTAACAGG
ATTAGCAGAGCGAGGTATGTAGGCGGTGCTACAGAGTTCTTGAAGTGGTGGCCTAACT
ACGGCTACACTAGAAGAACAGTATTTGGTATCTGCGCTCTGCTGAAGCCAGTTACCTTC
GGAAAAAGAGTTGGTAGCTCTTGATCCGGCAAACAAACCACCGCTGGTAGCGGTGGT
TTTTTTGTTTGCAAGCAGCAGATTACGCGCAGAAAAAAAGGATCTCAAGAAGATCCTT
TGATCTTTTCTACGGGGTCTGACGCTCAGTGGAACGAAAACTCACGTTAAGGGATTTT
GGTCATGAGATTATCAAAAAGGATCTTCACCTAGATCCTTTTAAATTAAAAATGAAGTT
TTAAATCAATCTAAAGTATATATGAGTAAACTTGGTCTGACAGTTACCAATGCTTAATCA
GTGAGGCACCTATCTCAGCGATCTGTCTATTTCGTTCATCCATAGTTGCCTGACTCCCCG

TCGTGTAGATAACTACGATACGGGAGGGCTTACCATCTGGCCCCAGTGCTGCAATGATA
CCGCGAGACCCACGCTCACCGGCTCCAGATTTATCAGCAATAAACCAGCCAGCCGGAA
GGGCCGAGCGCAGAAGTGGTCCTGCAACTTTATCCGCCTCCATCCAGTCTATTAATTGT
TGCCGGGAAGCTAGAGTAAGTAGTTCGCCAGTTAATAGTTTGCGCAACGTTGTTGCCAT
TGCTACAGGCATCGTGGTGTCACGCTCGTCGTTTGGTATGGCTTCATTCAGCTCCGGTT
CCCAACGATCAAGGCGAGTTACATGATCCCCCATGTTGTGCAAAAAAGCGGTTAGCTC
CTTCGGTCCTCCGATCGTTGTCAGAAGTAAGTTGGCCGCAGTGTTATCACTCATGGTTA
TGGCAGCACTGCATAATTCTCTTACTGTCATGCCATCCGTAAGATGCTTTTCTGTGACTG
GTGAGTACTCAACCAAGTCATTCTGAGAATAGTGTATGCGGCGACCGAGTTGCTCTTGC
CCGGCGTCAATACGGGATAATACCGCGCCACATAGCAGAACTTTAAAAGTGCTCATCAT
TGGAAAACGTTCTTCGGGGCGAAAACTCTCAAGGATCTTACCGCTGTTGAGATCCAGT
TCGATGTAACCCACTCGTGCACCCAACTGATCTTCAGCATCTTTTACTTTCACCAGCGT
TTCTGGGTGAGCAAAAACAGGAAGGCAAAATGCCGCAAAAAAGGGAATAAGGGCGA
CACGGAAATGTTGAATACTCATACTCTTCCTTTTTCAATATTATTGAAGCATTTATCAGG
GTTATTGTCTCATGAGCGGATACATATTTGAATGTATTTAGAAAAATAAACAAATAGGGG
TTCCGCGCACATTTCCCCGAAAAGTGCCACCTGACGTCGACGGATCGGGAGATCAACT
TGTTTATTGCAGCTTATAATGGTTACAAATAAAGCAATAGCATCACAAATTTCACAAATA
AAGCATTTTTTTCACTGCATTCTAGTTGTGGTTTGTCCAAACTCATCAATGTATCTTATC
ATGTCTGGATCAACTGGATAACTCAAGCTAACCAAAATCATCCCAAACTTCCCACCCCA
TACCCTATTACCACTGCCAATTACCTGTGGTTTCATTTACTCTAAACCTGTGATTCCTCT
GAATTATTTTCATTTTAAAGAAATTGTATTTGTTAAATATGTACTACAAACTTAGTAGT

Supplementary table 2. The primer sequences used for RT-PCR analysis

Gene name	Forward Primer Sequence (5’-3’)	Reverse Primer Sequence (5’-3’)
FUT8	GACAGAACTGGTTCAGCGGAGA	GCAGTAGACCACATGATGGAGC
BCL2A1	TACAGGCTGGCTCAGGACTAT	CGCAACATTTTGTAGCACTCTG
IL-8	CTTGGCAGCCTTCCTGA	TTCTTTAGCACTCCTTGGCAAAA
TRAF1	CGATGGCACTTTCCTGTGGAAG	TACAGCCGCAGGCACAACTTGT
TRAF2	GCTCATGCTGACCGAATGTC	GCCGTCACAAGTTAAGGGGAA
GADD45b	TGCTGTGACAACGACATCAAC	GTGAGGGTTCGTGACCAGG
ICAM1	ATGCCCAGACATCTGTGTCC	GGGGTCTCTATGCCCAACAA
PTGS2	ATGCTGACTATGGCTACAAAGC	TCGGGCAATCATCAGGC
c-IAP1	AGCACGATCTTGTCAGATTGG	GGCGGGGAAAGTTGAATATGTA
c-IAP2	AAGCTACCTCTCAGCCTACTTT	CCACTGTTTTCTGTACCCGGA
Bcl-xl	GCCACTTACCTGAATGACCACC	AACCAGCGGTTGAAGCGTTCCT
TNFAIP3	TCCTCAGGCTTTGTATITGAGC	TGTGTATCGGTGCATGGTITTA
TNFSF12	GAGGGGAAGGCTGTCTACCT	GAACCTGGAAGAGTCCGAAGTA
MAP3K14	AAAATGGCCCGTGTGTGTTG	GCCGAGTGGAGACTCATCC
MAP3K5	TTTTACCACCTTGGGGTGAGA	CTGTCACAGCAGTAGACTTTGTT
NFκBIA	CTCCGAGACTTTCGAGGAAATAC	GCCATTGTAGTTGGTAGCCTTCA
NFκB2	ATGGAGAGTTGCTACAACCCA	CTGTTCCACGATCACCAGGTA
RIPK2	GTGCCATTCACCTATGTGACA	GGACAGTGATGCAGCTTCATAAA
NGFR	CCTACGGCTACTACCAGGATG	CACACGGTGTTCTGCTTGT

Supplementary table 3. The antibody used for western blot analysis

Gene name	Manufacturer	Lot. No	Source	Dilution
FUT8	Affinity	DF13026	Rabbit	1:1000
Caspase 3	Affinity	DF6879	Rabbit	1:1000
Cleaved caspase 3	Affinity	AF7022	Rabbit	1:1000
PARP	Cell Signaling Technology	#9532	Rabbit	1:1000
Cleaved PARP	Affinity	AF7023	Rabbit	1:1000
Bcl-xl	Affinity	AF6414	Rabbit	1:1000
Bcl2A1	Affinity	DF7652	Rabbit	1:1000
Bcl-2	Affinity	AF6139	Rabbit	1:1000
TRAF-1	Affinity	AF5396	Rabbit	1:1000
Caspase8	SANTA ZRUZ BIOTECHNOLOGY	sc-81656	Mouse	1:1000
Cleaved Caspase 8	Affinity	AF5267	Rabbit	1:1000
Caspase 9	Cell Signaling Technology	9508	Mouse	1:1000
Cleaved caspase 9	Cell Signaling Technology	#Asp315	Rabbit	1:1000
NIK	Cell Signaling Technology	#4888	Rabbit	1:1000
Phospho-NF-κB2 p100	Cell Signaling Technology	#4888	Rabbit	1:1000
NF-κB2 p100/p52	Cell Signaling Technology	#4888	Rabbit	1:1000
PCNA	Affinity	AF0239	Rabbit	1:1000
FADD	SANTA ZRUZ BIOTECHNOLOGY	sc-271748	Mouse	1:1000
TRADD	Affinity	DF6279	Rabbit	1:1000
Tubulin beta	Affinity	AF7011	Rabbit	1:1000
GAPDH	Affinity	AF7021	Rabbit	1:1000
